# In vitro PCR verification that lysozyme inhibits nucleic acid replication and transcription

**DOI:** 10.1038/s41598-023-33228-6

**Published:** 2023-04-19

**Authors:** Lu Liu, Xu Jia, Xiaoyang Zhao, Ting Li, Ziren Luo, Ranxi Deng, Bijia Peng, Danting Mao, Hong Liu, Qian Zheng

**Affiliations:** 1 Medical Functional Experiment Center, North Sichuan Medical College, Nanchong, 637007 People’s Republic of China; 2 Department of Pharmacy, Affiliated Hospital of North Sichuan Medical College, Nanchong, 637000 People’s Republic of China

**Keywords:** Drug screening, Clinical pharmacology

## Abstract

Lysozyme can kill bacteria by its enzymatic activity or through a mechanism involving its cationic nature, which can facilitate electrostatic interactions with the viral capsid, the negatively charged parts of nucleic acids, and polymerase, so binding to nucleic acids may be another biological function of lysozyme. Here, PCR was used as a research tool to detect the effects of lysozyme on the replication and transcription of nucleic acids after treatment in different ways. We found that lysozyme and its hydrolysate can enter cells and inhibit PCR to varying degrees in vitro, and degraded lysozyme inhibited nucleic acid replication more effectively than intact lysozyme. The inhibition of lysozyme may be related to polymerase binding, and the sensitivity of different polymerases to lysozyme is inconsistent. Our findings provide a theoretical basis for further explaining the pharmacological effects of lysozyme, such as antibacterial, antiviral, anticancer, and immune regulatory activities, and directions for the development of new pharmacological effects of lysozyme and its metabolites.

## Introduction

All living things, including viruses, produce lysozymes, a group of naturally occurring alkaline enzymes that vary widely in their source, amount, structural, chemical, and enzymatic characteristics. Lysozymes are also known as muramidase or N-acetylmuramoylhydrolase^1^. In 1922, Fleming et al. isolated a protein from human bodily fluids and tears^2^ that can dissolve the cell walls of bacteria and named it lysozyme. Numerous organs can secrete lysozyme, which can be found in the mucous membrane, blood, secretory fluid, etc.^3,4^. In addition to being found in animals, lysozymes can also be found in the organs and secretions of numerous bacteria and plants^1^. Lysozyme can be divided based on source into plant type, animal type, phage type and bacterial type^5^. Animal lysozyme can also be divided into conventional, goose^6^ and i-type^7,8^. Lysozyme is stabilized by four disulfide links between its chain's eight cysteine residues^9^. The best source of this enzyme is chicken egg white^10^. As the initial research object of enzymology, lysozyme is an indispensable tool for cell fusion operations in the field of bioengineering and is considered to be one of the basic substances in the living world, of great significance for revealing basic mechanisms and life phenomena^11^.

Studies have shown that lysozyme has antimicrobial (e.g., bacterial, fungal and viral), anticancer, anti-inflammatory, immune regulation and other activities^12–14^. Much literature has reported the great potential of lysozymes to treat various types of pathogens in the clinical, feed and food fields^15^. The capacity of lysozyme to hydrolyze the β-1,4-glycosidic linkages between N-acetylmuramic acid and N-acetylglucosamide in the polysaccharide backbone of the peptidoglycans of the gram-positive bacterial cell wall makes it particularly effective against gram-positive bacteria. The mechanism of bacterial death may be related to the breakdown of insoluble mucopolysaccharides into soluble glycopeptides in the cell wall by lysozyme, leading to cell wall rupture and escape of the contents^16^. Due to the outer membrane's protective lipopolysaccharide layer, the action against gram-negative bacteria is noticeably weaker^17^. Through a mechanism involving its cationic nature, lysozyme can kill bacteria in addition to its enzyme activity and without the help of peptidoglycan breakdown. Human lysozyme and mouse LysM and LysP are examples of cationic C-type lysozymes. By inserting lysozyme into negatively charged bacterial membranes and creating pores, c-type lysozyme can kill bacteria through a process known as cationic killing. Therefore, the enzymatic and cationic characteristics of c-type lysozyme are related to its antibacterial activity^18–21^. However, most studies focus on the pharmacodynamic effects of lysozyme, with only hypotheses and inferences about the specific mechanisms.

Lysozyme is a key component of innate immunity and an essential component of host defense. However, the precise function of lysozyme in immune response defense is unclear, and the research methods and tools are complicated. Recent evidence has revealed the interesting immunomodulatory activity of lysozyme^22^, and the correlation between gonococcal susceptibility to lysozyme and increased neutrophil activation suggests the possibility that lysozyme may regulate immunological activation in other phagocytic cells. The Toll-like receptors (TLRs), inflammasomes, NOD1 and NOD2 receptors are all activated during lysozyme-mediated degradation^23,24^. Numerous cell types, including epithelial cells, express NOD1 widely, which supports proinflammatory signaling in these cell types^23^. On the one hand, the ability of lysozyme to breakdown PG changes the production of ligands that are identified by NODs^25,26^, which activates a variety of innate immune receptor families and triggers proinflammatory responses. NOD2-expressing cells are rare and mainly include phagocytes and some specialized cell types. Bacteria are phagocytosed and guided into lysosomes containing lysozyme and other antimicrobial components, according to the current working hypothesis for the activation of NOD family receptors in phagocytes^25^. Similar to macrophages, lysozyme-mediated destruction of phagocytic bacteria may enhance neutrophil activity^16^. However, insoluble PG but not soluble PG activates the inflammasome in macrophages, and inflammasome activation can also be triggered indirectly by the release of additional stimulatory bacterial substances by lysozyme. For instance, in vitro activation of inflammasomes by aureus lacking O-acetylation was decreased after inhibition of lysozyme^27^. However, it is unknown which PG structures NOD in phagocytes ultimately recognizes and how alterations that change lysozyme-mediated processing affect this identification. In addition to being the key to triggering the proinflammatory response, lysozyme also contributes to controlling systemic inflammation, which reduces inflammation-driven disease^18^. Studies have shown that lysozyme can reduce intestinal inflammation in animals with colitis induced by dextran sodium sulfate^28^. The mechanism may be related to the fact that lysozyme can help protect the intestinal epithelial barrier, release PG fragments that trigger protective intestinal immune responses, or remove polymeric PG that could hyperactivate local macrophages. In addition, exogenously added lysozyme reduced neutrophil chemotaxis and oxidative burst production by unknown mechanisms^29,30^. Finally, lysozyme can digest extracellular insoluble PGs into soluble fragments and reduce the production of anaphylaxis toxin and phagocytosis chemotaxis triggered by complement factors C3a and C5a, thereby reducing phagocytosis influx and accompanying cellular inflammation^31^. Taken together, these findings suggest a dual and potentially contradictory role for lysozyme in the immune response to infection. In-depth study of when, where and how lysozyme is released will illuminate the key mechanisms of lysozyme in host immune defense.

Based on the structural similarity between lysozyme and histones revealed by X-ray measurements, Steinrauf et al.^32^ speculated that lysozyme might have the potential to bind nucleic acids. Later, experiments using gel electrophoresis, enzyme activity, and coprecipitation have shown that binding to DNA and RNA is possible. Lee-Huang et al.^33^ found that RAWVAWRNR, a 9-peptide that constitutes human lysozyme, interferes with HIV-1 virus invasion and replication by affecting survival and stress-related pathways such as the TGFβ, p53, NF-κB, protein kinase C and hedgehog signaling pathways. However, it is still unclear how complete lysozyme and peptide fragments affect nucleic acid replication, as well as their specific targets. PCR was originally developed to detect mutations in the HBB gene that cause sickle cell anemia and was subsequently used for the analysis of multiallelic loci by hybridization of amplifiers to alleles^34,35^. Numerous clinical applications of PCR have followed, especially in the fields of bioscience, diagnostics and forensic science^36^. In this study, PCR was used as a research tool to detect whether lysozyme and its metabolites have inhibitory effects on the replication and transcription of nucleic acids in PCR and to provide a theoretical basis for further explaining the pharmacological effects of lysozyme, such as antibacterial, antiviral, anticancer, and immune regulation. Moreover, it provides guidance for the development of new pharmacological effects of lysozyme and its metabolites.

## Material and methods

### Plasmid construction, amplification and purification

#### Sequence construction

**Table:** 

Name	Sequence
Lentiviral vector-*EmGfp*_for	ATGGTGAGCAAGGGCGAGGAGCTGTTC
Lentiviral vector-*EmGfp*_rev	TTAGCTAGCCTTGTACAGCTCGTCCATG
Bacillus coli-*Amp*_for	ATGAGTATTCAACATTTCCGTGTCGC
Bacillus coli-*Amp*_rev	TTACCAATGCTTAATCAGTGAGGCACCTATC
Rat liver-*Grp78*_for	TGATTCCGAGGAACACTGTGG
Rat liver-*Grp78*_rev	CCCTTTGTCTTCAGCTGTCAC

All template DNAs were constructed on the GV657 universal expression vector plasmid containing the T7 promoter (all vectors were provided by Jikai Gene).

#### Plasmid DNA amplification and purification.

Plasmid DNA was amplified and purified using the TaKaRa MiniBEST Plasmid Purification Kit (9760-1, TaKaRa) according to the manufacturer’s instructions. The DNA concentration was measured with a NanoDrop Microvolume Spectrophotometer (Thermo Nanodrop 2000). A 1-μg sample of the plasmid DNA was linearized with 1 µl of ECORI (1040A, Takara), followed by phenol‒chloroform extraction and precipitation with ethanol. The samples were dissolved in RNase-free TE buffer and prediluted for quantitative real-time PCR as described below.

### Rat liver RNA extraction

Three Sprague‒Dawley (SD) rats weighing 200–220 g (HFK Bioscience, Beijing, China) were kept in our specific pathogen-free animal housing with a 12-h light/dark cycle. All rats had free access to sterilized water and standard rodent chow and resided in sterilized cages housed in laminar flow hoods. All procedures were approved by the Institute Animal Care and Use Committee of the North Sichuan Medical College (Nanchong, China). All animal experiments were performed in accordance with the relevant guidelines and regulations, and the animal study is reported in accordance with ARRIVE guidelines. Rats were euthanized by intraperitoneal injection of pentobarbital sodium (30 mg/kg) after 30 days of feeding, and total RNA was isolated from 200 mg of liver tissue of each rat using the classic TRIzol (Life Invitrogen) method. The RNA concentration was measured with a NanoDrop Microvolume Spectrophotometer and stored at − 80 °C.

### Cell culture and treatment

HepG2 cells were purchased from the Procell Company (China) and cultured in Dulbecco’s minimal essential medium (DMEM) (HyClone). Experiments were performed when cells reached 70–80% confluency. Briefly, HepG2 cells (5 × 10^5^ cells/mL) were seeded in triplicate in 6-well plates overnight and randomly divided into 2 groups. One group of cells continued to be cultured for 24 h. Then, sonication was performed to disrupt the cells using defined conditions (25 mV, 10 min, 4 °C). The cell lysate supernatant was collected after centrifugation (12,000 rpm, 5 min, 4 °C) and subsequently incubated with 50 mg/ml lysozyme (*n* = 3) for 1 h at 37 °C. Another group of cells was cocultured with lysozyme according to the above concentration gradient (*n* = 3) for 24 h at 37 °C. The cell culture supernatant containing lysozyme and cells was collected separately, the culture supernatant was centrifuged at 10,000 rpm for 5 min, and the precipitate was discarded. The cocultured cells were disrupted by low-temperature ultrasound and centrifuged according to the above method. All cocultivation liquid was then collected for subsequent analysis.

### Artificial gastric juice preparation and incubation with lysozyme

Artificial gastric juice was prepared with reference to the Chinese Pharmacopoeia 2015. The lysozyme solution was pipetted into a 10-ml sterile centrifuge tube and diluted to 8 ml with artificial gastric juice for a final lysozyme concentration of 10 mg/ml (*n* = 3). The experimental group (*n* = 3) was incubated at 37 °C for 1 h, the control group (*n* = 3) was centrifuged (4 °C, 12,000 r/min, 10 min), and the supernatant was collected immediately after adding the lysozyme solution. The supernatant was then stored at − 80 °C for subsequent analysis.

### Rabbit liver tissue lysate incubated with lysozyme

Three rabbits per treatment were sacrificed by pentobarbital overdose (30 mg/kg bodyweight). All procedures were approved by the Institute Animal Care and Use Committee of the North Sichuan Medical College (Nanchong, China). All animal experiments were performed in accordance with the relevant guidelines and regulations, and the animal study is reported in accordance with ARRIVE guidelines. The thorax was opened, and 200 mg of liver tissue was collected from each rabbit. After being snap-frozen in liquid nitrogen, the liver samples were dissected and lysed with cold PBS (HyClone) and then centrifuged (4 °C, 12,000 r/min, 10 min), and the supernatant was collected. Protein concentrations were measured using a BCA Assay Kit (Beyotime Biotech) and normalized to 10 mg/ml lysozyme solution. The experimental group (*n* = 3) was coincubated with 10 mg/ml lysozyme at 37 °C for 1 h. The control group (*n* = 3) was centrifuged (4 °C, 12,000 r/min, 10 min), and the supernatant was collected immediately after adding the lysozyme solution. The supernatant was then stored at − 80 °C for subsequent analysis.

### Rabbit gut lavage fluid and fecal lavage fluid incubated with lysozyme

To stimulate the secretion of digestive juices, three rabbits were fasted for 12 h with free access to water and then fed a small amount of fodder (~ 50 g) half an hour before sampling. After the rabbit was killed by pentobarbital overdose (30 mg/kg bodyweight), pieces of the rabbit jejunum, ileum and colon were removed, followed by lavage with PBS (37 °C). The gut lavage fluid was subsequently coincubated with 50 mg/ml lysozyme solution (the final volume of the solution was adjusted to 40 ml with PBS). Another three rabbits were sacrificed according to the above method. The feces of the rabbit sigmoid colon and rectum were collected, washed with PBS and filtered through gauze. The filtrate was subsequently coincubated with 50 mg/ml lysozyme solution (the final volume of the solution was adjusted to 40 ml with PBS). The experimental group (*n* = 3) was incubated at 37 °C for 1 h, the control group (*n* = 3) was centrifuged (4 °C, 12,000 r/min, 10 min), and the supernatant was collected immediately after adding the lysozyme solution. The supernatant was then stored at − 80 °C for subsequent analysis.

### Lysozyme API and ECT gavage

Rabbits were randomly divided into the control group, the active pharmaceutical ingredient (API) group and the enteric coated tablet (ECT) group, with 3 rabbits in each group. After weighing, 30 mg/kg sodium pentobarbital was used to induce anesthesia. The rabbits were treated with lysozyme API (180 mg/kg) and lysozyme ECT by gavage (180 mg/kg). The control group was given the corresponding volume of vehicle or placebo. After 1 h of intervention, the rabbits were treated as described above, and the gastric, jejunum and ileum lavage fluids were centrifuged (4 °C, 12,000 r/min, 10 min). The supernatant was collected for subsequent PCR analysis.

### DNA‒DNA amplification system

To investigate the effect of lysozyme on the amplification system of EmGfp, Amp and Grp78 templates, coincubation solution containing lysozyme pretreated under different conditions was added to the PCR system (diluted lysozyme to the corresponding working concentration). For qPCR, SYBR green (TAKARA) was used along with the Real-Time PCR System (ABI7500, USA). Thermal cycles included 15 min at 94 °C followed by 25 cycles of 20 s at 94 °C, 20 s at 60 °C, and 90 s at 72 °C and finally 5 min at 72 °C. The amplified PCR products were evaluated by 1.5% agarose gel electrophoresis in Tris–borate–EDTA buffer stained with ethidium bromide. Band abundance was used as the criterion for amplification efficiency. All qPCR analysis reactions were performed in triplicate on at least two biological replicates.

### DNA‒RNA transcription system

To investigate the effect of lysozyme on the transcription system of EmGfp, Amp and Grp78 templates, transcribed RNA sequences were obtained using an in vitro Transcription T7 Kit (Takara Bio, 6140). The in vitro transcription reaction solution was prepared by mixing 17 μL of T7 transcription kit solution, 1 μL of 1 μg/μL template DNA and 2 μL of treatment solution containing lysozyme. The reaction solution was incubated at 42 °C for 1–2 h. After incubation, 10–20 U RNase-free DNase I (Takara Bio) was added, and the reaction solution was incubated at 37 °C for 30 min to remove DNase. RNA fragments were extracted using a NucleoSpin RNA Clean-up Kit (Takara Bio, 740,948.250) according to the manufacturer’s protocol. One microgram of total RNA was reverse transcribed using the PrimeScript RT reagent kit with gDNA Eraser (Takara Bio, 047A), and cDNA was stored at − 20 °C for subsequent PCR analysis. For qPCR, thermal cycles included 5 min at 94 °C followed by 25 cycles of 20 s at 94 °C, 20 s at 60 °C, and 90 s at 72 °C and finally 5 min at 72 °C. The amplified PCR products were evaluated by 1.5% agarose gel electrophoresis in Tris–borate–EDTA buffer stained with ethidium bromide. Band abundance was used as the criterion for amplification efficiency. All qPCR analysis reactions were performed in triplicate on at least two biological replicates.

### RNA‒DNA reverse transcription system

In vitro transcription and RNA purification were performed according to the above methods, and then different concentration gradients of lysozyme were added to the reverse transcription system to study the effect of lysozyme on the reverse transcription process. The mixture was incubated at 37 °C for 15 min and then kept at 85 °C for 10 s, and cDNA was stored at − 20 °C for subsequent PCR analysis. The Q-PCR and agarose gel electrophoresis methods are described in DNA‒RNA transcription system.

### Statistical analysis

Data were expressed as the mean ± standard deviation and analyzed using SPSS 22.0 statistical software for all groups following a normal distribution. The F test was used to compare the means of multiple samples. Dunnett’s t test and the SNK-q test were used for multiple comparisons between multiple sample means. A value of *p* < 0.05 was considered to indicate a significant difference.

### Ethical approval

All procedures were approved by the Institute Animal Care and Use Committee of the North Sichuan Medical College (Nanchong, China). All animal experiments were performed in accordance with the relevant guidelines and regulations, and the animal study is reported in accordance with ARRIVE guidelines.

## Results

### Lysozyme shows a potent and reproducible dose-dependent inhibitory effect on DNA replication, transcription and reverse transcription in vitro

**Figure 1:**
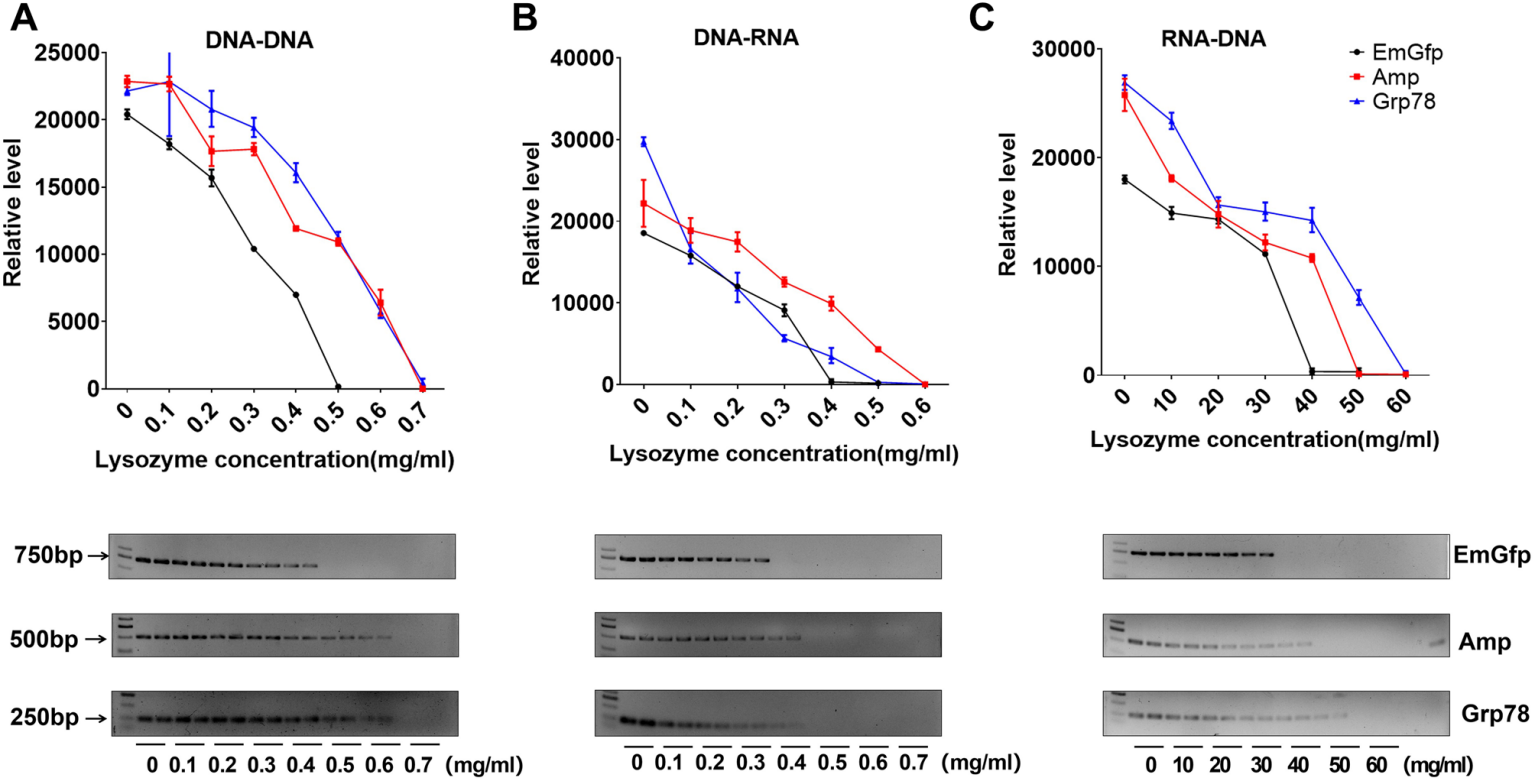
Lysozyme blocks EmGfp, Amp and Grp78 DNA replication, transcription and reverse transcription. (**A**)**–**(**C**) Gel electrophoresis of PCR products. Lysozyme at the corresponding working concentration was added to each reaction system. (**A**) Replication, (**B**) transcription and (**C**) reverse transcription. Intensity analysis of target bands in gel electrophoresis is shown at the top of the blot, original blots/gels are presented in Supplementary data (PCR-full-length immunoblots). Data are representative of 2 independent experiments and presented as the mean ± SD; *p < 0.05, ***p < 0.001. Significant differences were evaluated using one-way ANOVA with a post hoc test (Fisher’s least significant difference).

To investigate the dose–effect relationship of lysozyme on the RT‒PCR system of lentiviral (EmGfp), Bacillus colony (Amp) and rat liver (Grp78) genes, lysozyme was added to each PCR system at the corresponding working concentration. The products obtained through PCR were compared using gel electrophoresis. By semiquantitative analysis, with intensity measured using ImageJ, we observed that lysozyme had a potent and reproducible dose-dependent inhibitory effect on DNA replication, transcription and reverse transcription in vitro (Fig. 1A–C). The replication and transcription of the three genes showed sensitivity to low concentrations of lysozyme, while almost tenfold levels of lysozyme could obviously inhibit the reverse transcription reaction. Significant inhibition was observed above 0.5 mg/ml lysozyme in the replication of EmGfp and transcription of Amp and Grp78 DNA. Lysozyme significantly blocked Amp and Grp78 replication at 0.7 mg/ml and EmGfp transcription at 0.4 mg/ml (Fig. 1A,B). For reverse transcription, lysozyme treatment caused a drop of more than 90% in the products of EmGfp, Amp and Grp78 at 40 mg/ml, 50 mg/ml, and 50 mg/ml, respectively (Fig. 1C).

### The mixture of lysozyme and cell lysate after coincubation for 1 h showed a stronger dose-dependent inhibitory effect on the PCR process in vitro

**Figure 2:**
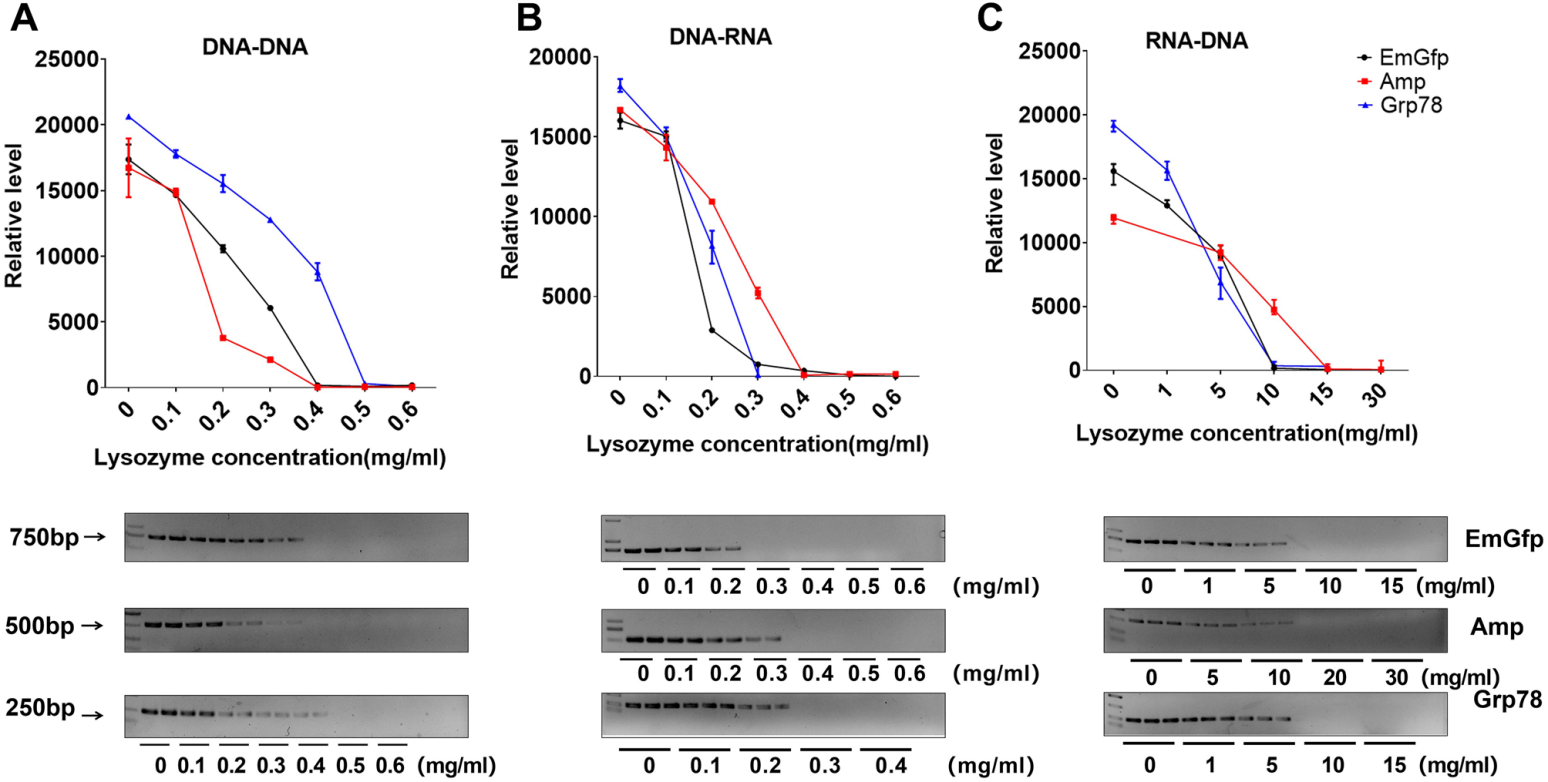
Cell lysate-treated lysozyme blocks EmGfp, Amp and Grp78 DNA replication, transcription and reverse transcription. (**A**)–(**C**) Gel electrophoresis of PCR products. Cell lysate-treated lysozyme at the corresponding working concentration was added to the reaction system: (**A**) replication, (**B**) transcription and (**C**) reverse transcription. Intensity analysis of target bands in gel electrophoresis is shown at the top of the blot. Data are representative of 2 independent experiments and presented as the mean ± SD; *p < 0.05, ***p < 0.001. Significant differences were evaluated using one-way ANOVA with a post hoc test (Fisher’s least significant difference).

To determine the dose–effect relationship of lysozyme after coincubation with HepG2 cell lysate supernatant for 1 h in a PCR system of lentiviral (EmGfp), Bacillus col (Amp) and rat liver (Grp78) genes, lysozyme at the corresponding working concentration was added to the PCR system as previously described. The products obtained through PCR were compared using gel electrophoresis. For semiquantitative analysis, the intensity was measured using ImageJ. Consistent with the above results, the mixture of cell lysate and lysozyme inhibited DNA replication, transcription and reverse transcription in a dose-dependent manner in vitro. We noticed that compared with adding lysozyme directly to the PCR system, the mixture of lysozyme and cell lysate after coincubation for 1 h showed a stronger inhibitory effect on the PCR process. Significant inhibition of the replication of EmGfp and Amp and the transcription of Amp DNA was observed above 0.4 mg/ml cell lysate-treated lysozyme. Cell lysate-treated lysozyme blocked EmGfp and Grp78 replication at 0.3 mg/ml and Grp78 transcription at 0.5 mg/ml (Fig. 2A,B). Notably, for reverse transcription, cell lysate-treated lysozyme caused a drop of more than 90% in the products of EmGfp, Amp and Grp78 at 10 mg/ml, 20 mg/ml, and 10 mg/ml, respectively (Fig. 2C).

### After coculture of HepG2 cells with lysozyme for 24 h, the cell lysate and supernatant of HepG2 cells showed different effects on the PCR process in vitro

**Figure 3:**
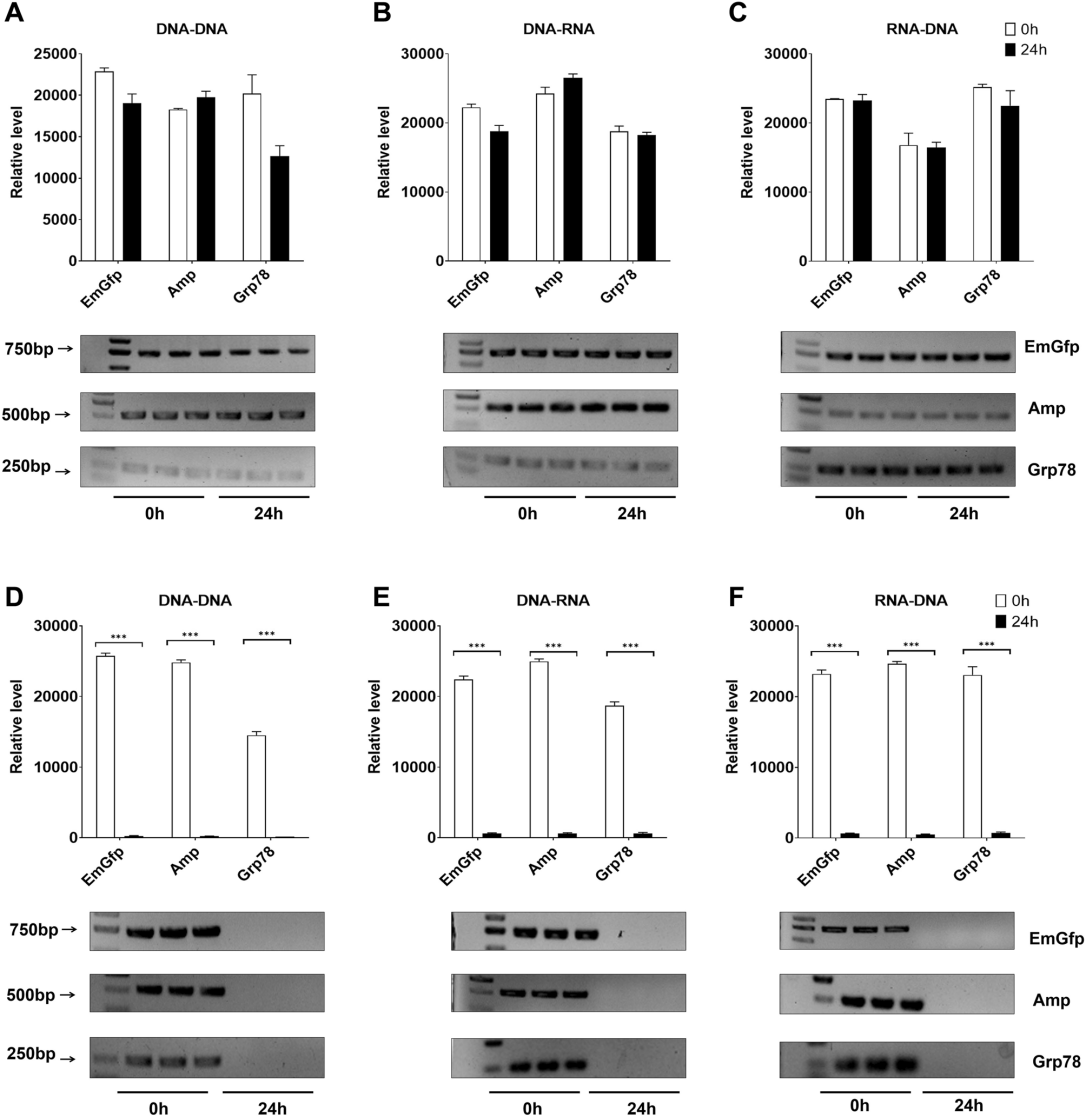
After coculture of HepG2 cells with lysozyme for 24 h, the cell lysate and supernatant of HepG2 cells showed different effects on the PCR process in vitro. (**A**)–(**F**) Gel electrophoresis of PCR products. The supernatant (**A**)–(**C**) and cell lysate (**D**)–(**F**) of HepG2 cells were added to the reaction system: (**A**/**D**) replication, (**B**/**E**) transcription and (**C**/**F**) reverse transcription. Intensity analysis of target bands in gel electrophoresis results shown to the top of the blot. Data are representative of 2 independent experiments and presented as the mean ± SD; *p < 0.05, ***p < 0.001. Significant differences were evaluated using one-way ANOVA with a post hoc test (Fisher’s least significant difference).

Among the concentrations tested, the inhibitory effect of lysozyme on DNA replication, transcription and reverse transcription increased after incubation in cell lysate. To further evaluate the effect of lysozyme on the PCR system and to ensure that PCR products could be observed in subsequent experiments, we chose a series of concentrations of lysozyme for coculture with HepG2 cells for 24 h according to the above experimental results and then took the cell culture supernatant and cell lysate and added them to the PCR system. Compared with the control, the cell culture supernatant obtained from HepG2 cells cocultured with 0.3–0.5 mg/ml, 0.3–0.4 mg/ml, or 10–20 mg/ml lysozyme did not show changes in DNA replication, transcription or reverse transcription (Fig. 3A–C). Finally, we determined the test concentrations of the three genes in the process of transcription, translation, and reverse transcription. When compared to the unincubated control, culturing HepG2 cells in lysozyme conditions for 24 h markedly decreased the fraction of products obtained by PCR (Fig. 3D–F). Briefly, significant inhibition of the replication of EmGfp and transcription of EmGfp and Grp78 DNA was observed when cell lysate cocultured with 0.3 mg/ml lysozyme was added to the PCR system. Cell lysate blocked Amp and Grp78 replication when cocultured with 0.5 mg/ml lysozyme and Grp78 transcription at 0.4 mg/ml (Fig. 3D–E). Notably, for reverse transcription, cell lysate cocultured with lysozyme caused more than a 90% drop in the products of EmGfp, Amp and Grp78 at 10 mg/ml, 20 mg/ml, and 10 mg/ml, respectively (Fig. 3F).

### Artificial gastric juice and rabbit liver tissue lysate-treated lysozyme inhibit the PCR process in vitro

**Figure 4:**
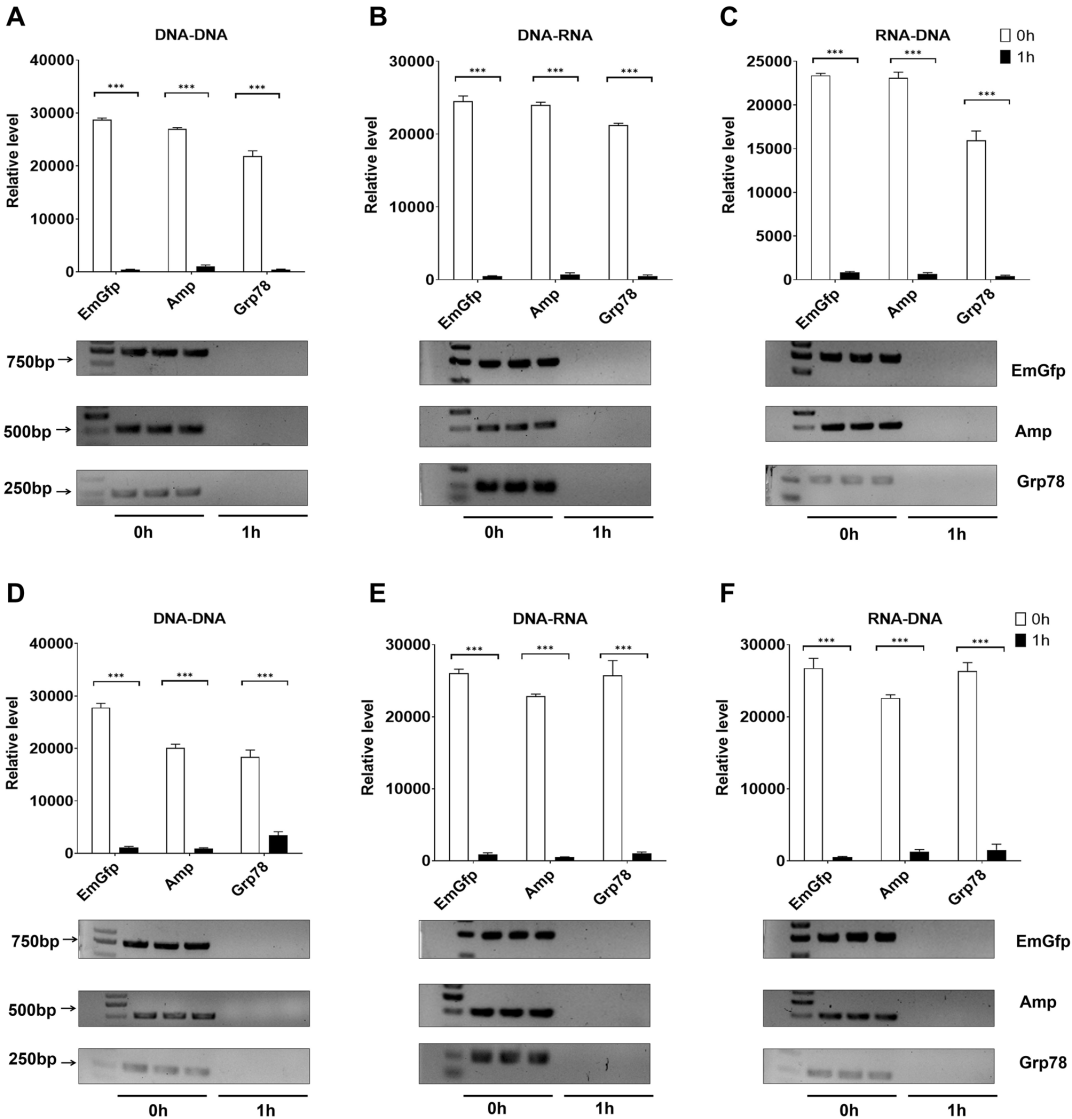
Artificial gastric juice and rabbit liver tissue lysate-treated lysozyme blocks EmGfp, Amp and Grp78 DNA replication, transcription and reverse transcription in vitro. (**A**)–(**F**) Gel electrophoresis of PCR products. Artificial gastric juice-treated lysozyme (**A**)–(**C**) and rabbit liver tissue lysate-treated lysozyme (**D**)–(**F**) were added to the reaction system: (**A**/**D**) replication, (**B**/**E**) transcription and (**C**/**F**) reverse transcription. Intensity analysis of target bands in gel electrophoresis is shown at the top of the blot. Data are representative of 2 independent experiments and presented as the mean ± SD; *p < 0.05, ***p < 0.001. Significant differences were evaluated using one-way ANOVA with a post hoc test (Fisher’s least significant difference).

We next assayed the effects of gastric juice and liver tissue lysate-treated lysozyme on the PCR system. Compared with the control, lysozyme incubated with artificial gastric juice for 1 h markedly decreased the fraction of products obtained by PCR (Fig. 4A–C). Briefly, significant inhibition of the replication of EmGfp and transcription of EmGfp and Grp78 DNA was observed when lysozyme (final concentration 0.3 mg/ml) was added to the PCR system after incubation with artificial gastric juice. Coincubation with artificial gastric juice and lysozyme blocked Amp and Grp78 replication (final concentration 0.5 mg/ml) and Grp78 transcription (final concentration 0.4 mg/ml) (Fig. 4A,B). Notably, for reverse transcription, the coincubation of artificial gastric juice and lysozyme caused a drop of more than 90% in the products of EmGfp, Amp and Grp78 at 10 mg/ml, 20 mg/ml, and 10 mg/ml, respectively (Fig. 4C). Consistent with the inhibitory effect of the coincubated solution of artificial gastric juice and lysozyme on PCR, using the concentrations tested in this assay, the coincubation solution of rabbit liver tissue lysate and lysozyme exhibited a significant inhibitory effect on EmGfp, Amp and Grp78 DNA replication, transcription and reverse transcription compared with the unincubated control (Fig. 4D–F).

### Rabbit jejunum lavage fluid treatment somewhat enhances the inhibitory effect of lysozyme on replication, transcription and reverse transcription of EmGfp, Amp and Grp78 DNA

**Figure 5:**
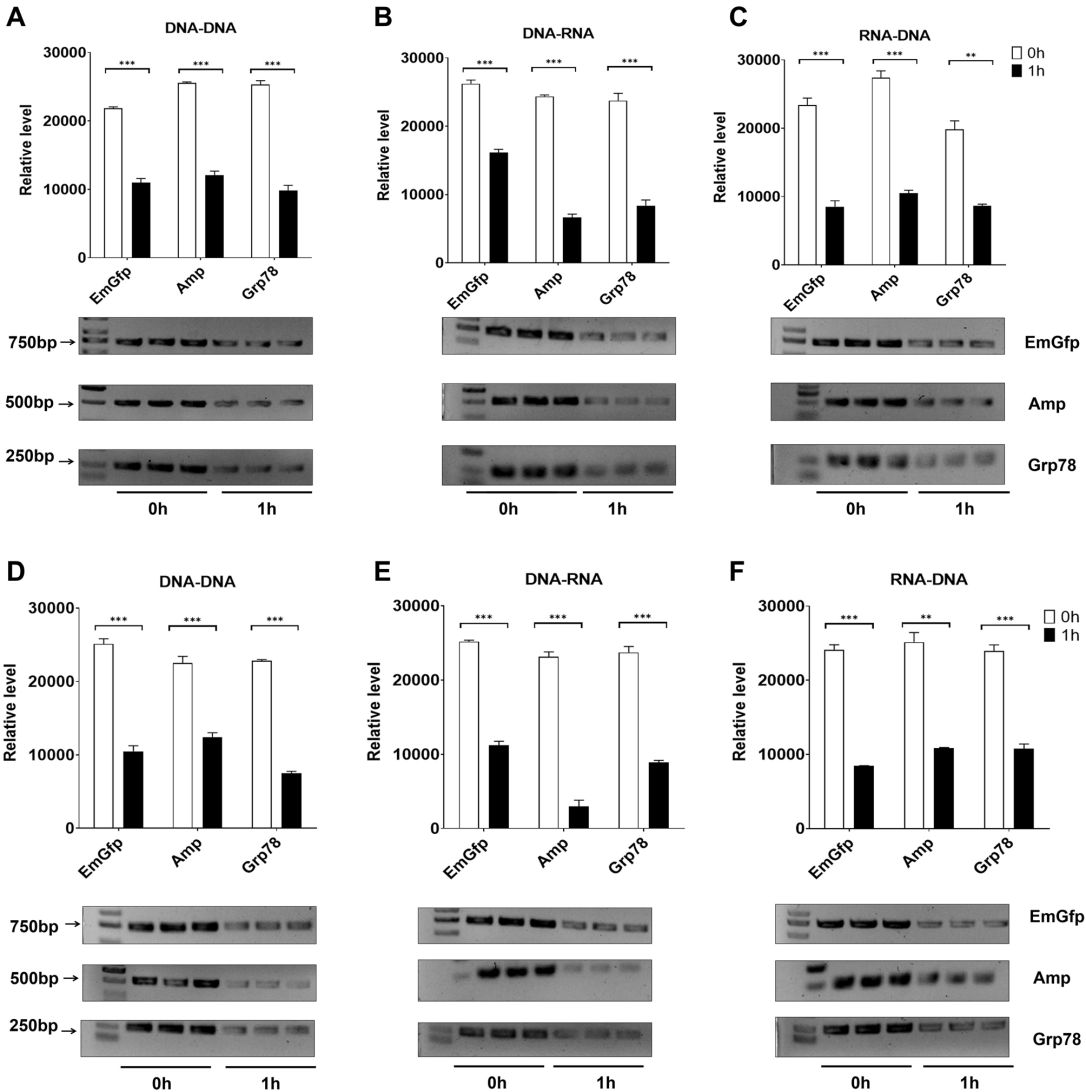
Rabbit jejunum lavage fluid-treated lysozyme partially blocks EmGfp, Amp and Grp78 DNA replication, transcription and reverse transcription in vitro. (**A**)–(**F**) Gel electrophoresis of PCR products. Lysozyme treated with lavage fluid of the upper (**A**)–(**C**) and lower (**D**)–(**F**) jejunum was added to the reaction system: (**A**/**D**) replication, (**B**/**E**) transcription and (**C**/**F**) reverse transcription. Intensity analysis of target bands in gel electrophoresis is shown at the top of the blot. Data are representative of 2 independent experiments and presented as the mean ± SD; *p < 0.05, ***p < 0.001. Significant differences were evaluated using one-way ANOVA with a post hoc test (Fisher’s least significant difference).

We next examined the effects of rabbit jejunum and ileum lavage fluid-treated lysozyme on the PCR system. Compared with the control, lysozyme incubated with lavage fluid of the upper and lower jejunum for 1 h somewhat decreased the fraction of products obtained by PCR (Fig. 5A–E). Briefly, an approximately onefold reduction was observed in the replication of EmGfp and transcription of EmGfp and Grp78 DNA when lysozyme (final concentration 0.3 mg/ml) was added to the PCR system after incubation with jejunum lavage fluid. Coincubation with jejunum lavage fluid and lysozyme partially blocked Amp and Grp78 replication (final concentration 0.5 mg/ml) and Grp78 transcription (final concentration 0.4 mg/ml) (Fig. 5A–D). Notably, for reverse transcription, the coincubation of jejunum lavage fluid and lysozyme caused a drop of more than 50^ in the products of EmGfp, Amp and Grp78 at 10 mg/ml, 20 mg/ml, and 10 mg/ml, respectively (Fig. 5E,F). However, inconsistent with the inhibition of PCR by the coincubation solution of jejunum lavage fluid and lysozyme using the concentrations tested in this assay, the coincubation solution of lavage fluid of the upper or lower ileum and lysozyme did not exhibit inhibitory effects on EmGfp, Amp and Grp78 DNA replication, transcription and reverse transcription compared with the unincubated control (Fig. S1). Notably, rabbit colon lavage fluid (Fig. S2) and fecal filtrate (Fig. S3)-treated lysozyme at the concentrations tested also had no effect on EmGfp, Amp and Grp78 DNA replication, transcription or reverse transcription in vitro.

### Effects of gastrointestinal lavage fluid on PCR after oral administration of lysozyme API in rabbits

**Figure 6:**
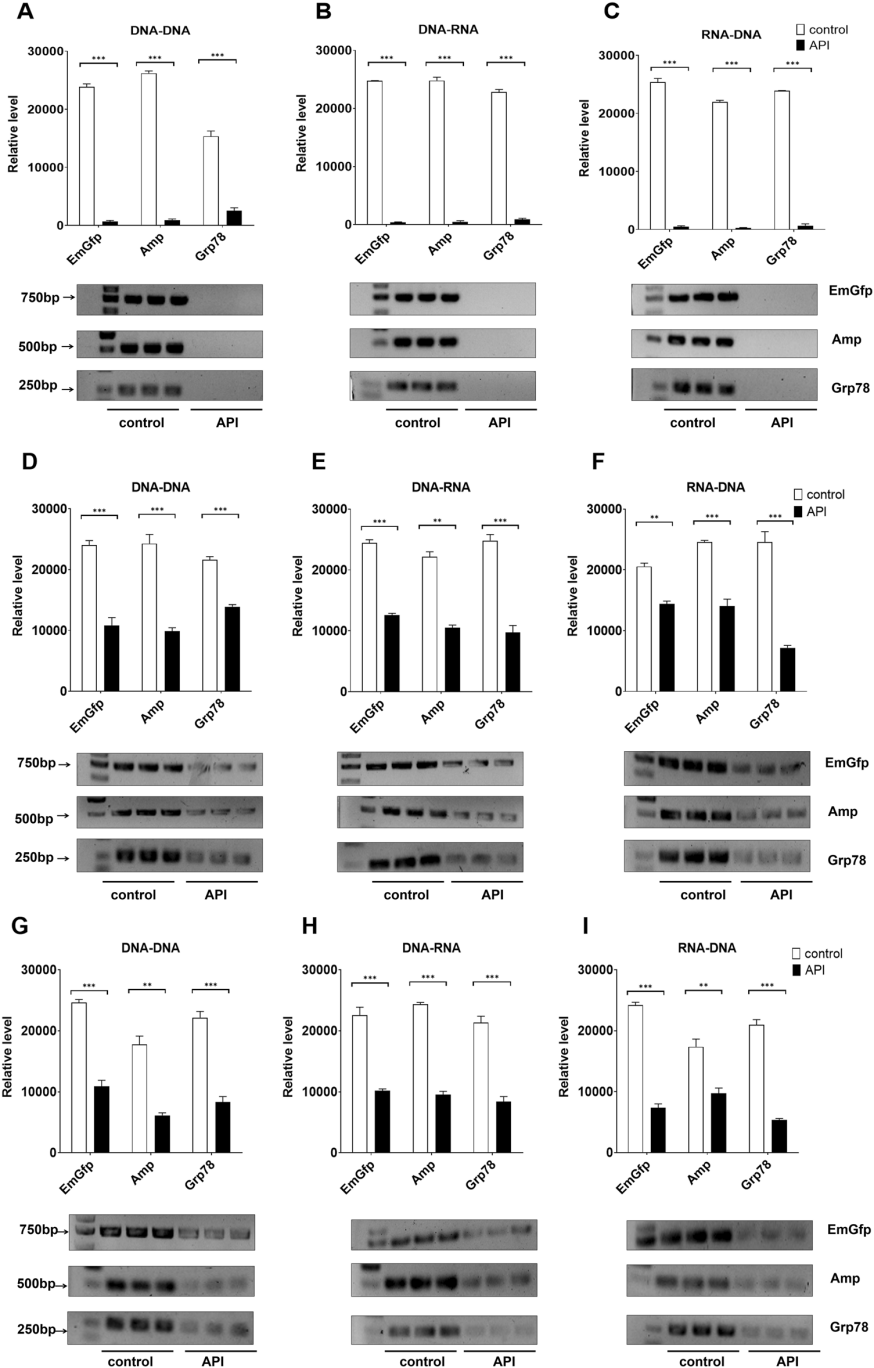
After lysozyme API gavage, rabbit gastric juice and jejunum fluid alter EmGfp, Amp and Grp78 DNA replication, transcription and reverse transcription in vitro. (**A**)–(**I**) Gel electrophoresis of PCR products. Lavage fluid of the gastric juices (**A**)–(**C**) and the upper (**D**)–(**F**) and lower (**G**)–(**I**) jejunum were added to the reaction system: (**A**/**D**/**G**) replication, (**B**/**E**/**H**) transcription and (**C**/**F**/**I**) reverse transcription. Intensity analysis of target bands in gel electrophoresis is shown at the top of the blot. Data are representative of 2 independent experiments and presented as the mean ± SD; *p < 0.05, ***p < 0.001. Significant differences were evaluated using one-way ANOVA with a post hoc test (Fisher’s least significant difference).

Because there was an enhanced inhibitory effect on DNA replication, transcription and reverse transcription after lysozyme was coincubated with gastrointestinal lavage fluid, we next examined whether rabbit gastrointestinal lavage fluid alters PCR after the administration of lysozyme API by gavage. The control group was given the corresponding volume of vehicle or placebo. At the tested lysozyme concentration, gastric juice markedly decreased the fraction of products obtained by PCR (Fig. 6A–C) compared with the control. Consistent with previous observations, significant inhibition of the replication of EmGfp and transcription of EmGfp and Grp78 DNA was observed when gastric juice (gavage concentration of lysozyme API 0.3 mg/ml) was added to the PCR system. Gastric juice blocked Amp and Grp78 replication at 0.5 mg/ml lysozyme API gavage and Grp78 transcription at 0.4 mg/ml lysozyme API gavage (Fig. 6A,B). Notably, for reverse transcription, gastric juice caused a drop of more than 90% in the products of EmGfp, Amp and Grp78 at 10 mg/ml, 20 mg/ml, and 10 mg/ml lysozyme API gavage, respectively (Fig. 6C). Consistent with the inhibition of PCR by the coincubation solution of jejunum lavage fluid and lysozyme using the concentrations tested in this assay, lavage fluid from the upper and lower jejunum somewhat decreased the fraction of products obtained by PCR after lysozyme API gavage (Fig. 6D–I). Notably, consistent with the assumptions, after lysozyme API gavage, neither the upper nor lower ileum fluid caused altered levels of EmGfp, Amp and Grp78 DNA replication, transcription and reverse transcription compared with the control (Fig. S4).

### Effects of gastrointestinal lavage fluid on PCR after oral administration of lysozyme ECT in rabbits

**Figure 7:**
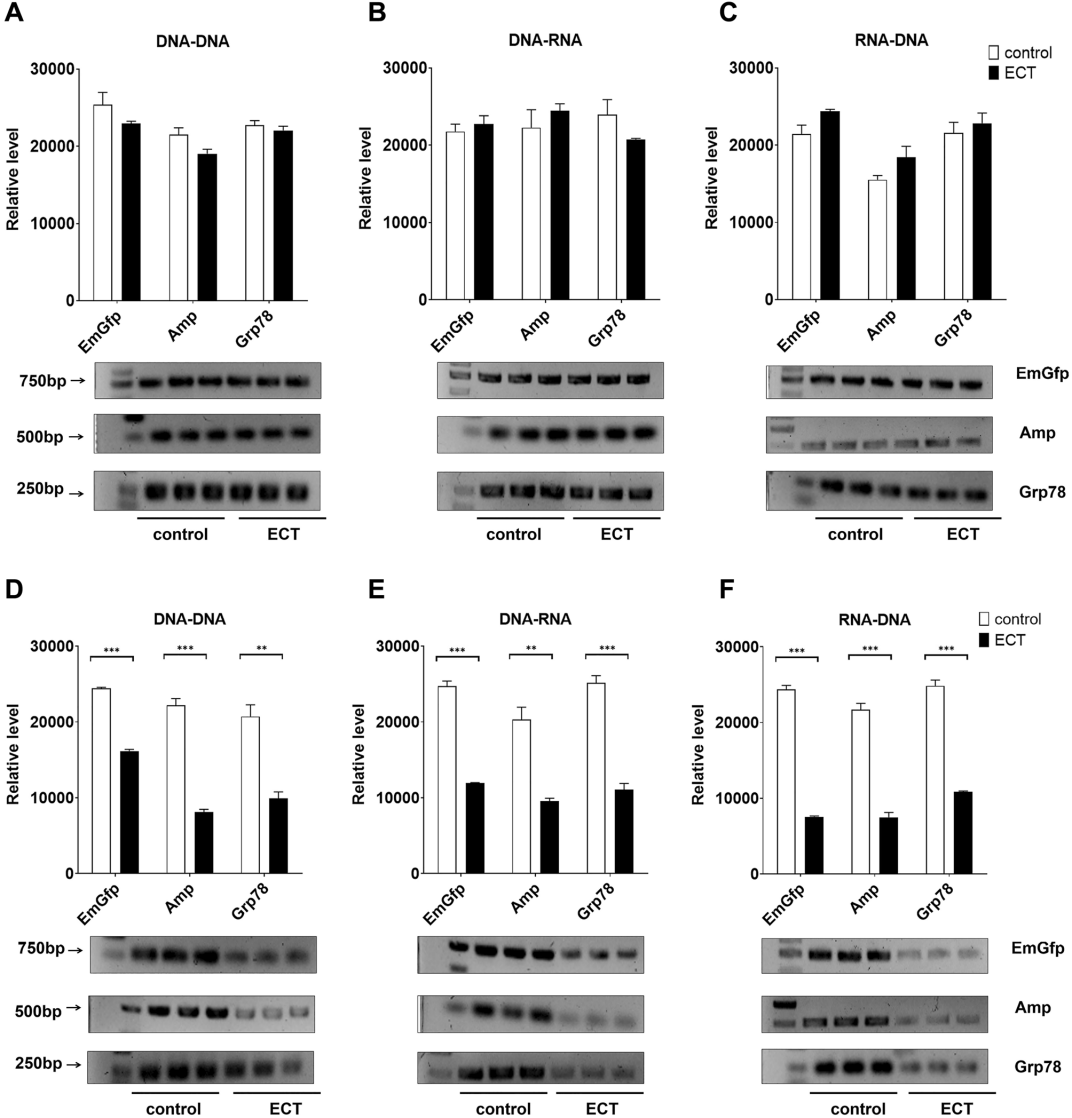

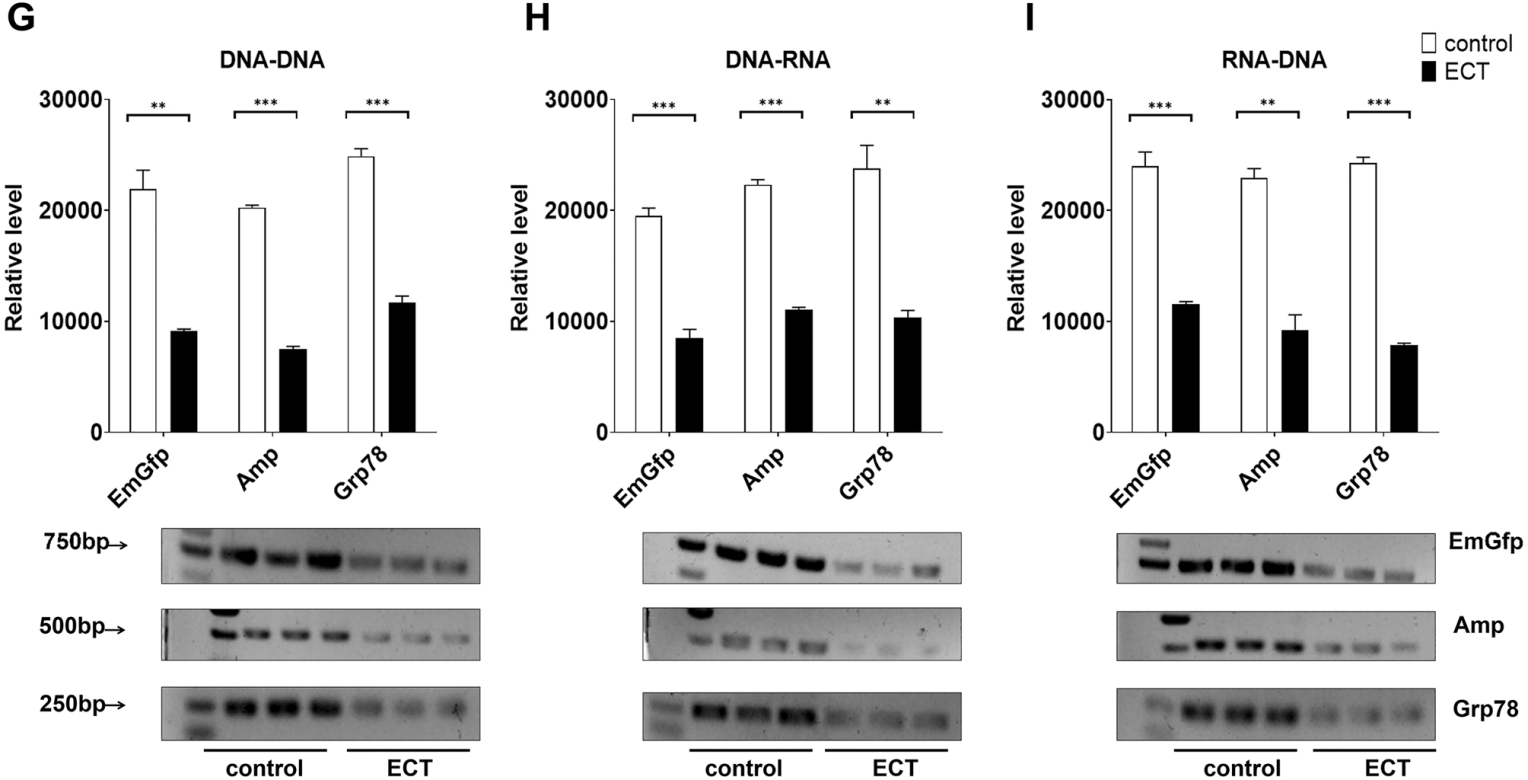
After lysozyme ECT gavage, rabbit gastric juice and jejunum fluid showed different effects on EmGfp, Amp and Grp78 DNA replication, transcription and reverse transcription in vitro. (**A**)–(**I**) Gel electrophoresis of PCR products. Lavage fluid of the gastric juice (**A**)–(**C**) and the upper (**D**)–(**F**) and lower (**G**)–(**I**) jejunum were added to the reaction system: (**A**/**D**/**G**) replication, (**B**/**E**/**H**) transcription and (**C**/**F**/**I**) reverse transcription. Intensity analysis of target bands in gel electrophoresis is shown at the top of the blot. Data are representative of 2 independent experiments and presented as the mean ± SD; *p < 0.05, ***p < 0.001. Significant differences were evaluated using one-way ANOVA with a post hoc test (Fisher’s least significant difference).

We next examined whether rabbit gastrointestinal lavage fluid alters PCR after the administration of lysozyme ECT by gavage. The control group was given the corresponding volume of vehicle or placebo. However, at the tested lysozyme concentration, we found that gastric juice had no observable effect on the fraction of products obtained by PCR (Fig. 7A–C) compared with the control. Notably, consistent with lysozyme API gavage partially inhibiting PCR, using the concentrations tested in this assay, the upper and lower jejunum partially decreased the fraction of products obtained by PCR after lysozyme ECT gavage (Fig. 7D–I). Briefly, an approximately onefold reduction was observed in the replication of EmGfp and the transcription of EmGfp and Grp78 DNA when jejunum fluid (gavage concentration of lysozyme ECT 0.3 mg/ml) was added to the PCR system. The jejunum fluid partially blocked Amp and Grp78 replication at 0.5 mg/ml lysozyme ECT gavage and Grp78 transcription at 0.4 mg/ml lysozyme ECT gavage. Notably, for reverse transcription, the jejunum fluid caused a drop of more than 50% in the percent products of EmGfp, Amp and Grp78 at 10 mg/ml, 20 mg/ml, and 10 mg/ml lysozyme ECT gavage, respectively (Fig. 7D–I). Consistent with previous observations, after lysozyme ECT gavage, neither the upper nor lower ileum fluid altered the levels of EmGfp, Amp and Grp78 DNA replication, transcription and reverse transcription compared with the control (Fig. S5).

## Discussion

The majority of current drug discovery research has focused on the analysis of interactions between chemical molecules and biological targets. Approximately 10,000 druggable targets may exist, according to genomic studies; however, only 5% of these targets have matching FDA-approved medicines^37^. Therefore, one of the most difficult jobs for modern drug development is to find drug candidates quickly and cheaply. At present, PCR is only used as a scientific research tool for qualitative or quantitative nucleic acid detection^38^ and has historically been underutilized as a screening tool for antiviral, gene inhibition, antitumor and other active drugs. Lysozyme is a natural remedy that offers the benefits of good tolerance and the absence of adverse effects. The interaction of lysozyme with other proteins and signaling pathways has been the subject of numerous studies, although the mechanism is still unclear. Lysozyme is a heat-resistant enzyme that can maintain a stable chemical structure within a certain pH range (3–7). Lysozyme still maintains good bioactivity at 100 °C in acidic solution (pH = 3)^39,40^. Based on its thermostability, lysozyme can exhibit partial biological activities at PCR temperatures. Meanwhile, lysozyme is a weakly basic protein with some basic amino acid residues. The lysozyme reaction system consists of PCR buffer (pH ≈ 7.5), so its biological activity is theoretically stable in the PCR system. In this study, we added lysozyme and its metabolites to the PCR system and analyzed the quantity of PCR products by gel electrophoresis. It was preliminarily observed that lysozyme and its metabolites inhibited PCR to varying degrees in vitro. Our study not only expands the utility of PCR as a screening platform and tool for molecular biology but also illuminates the mechanism by which lysozyme inhibits nucleic acid replication.

However, the peptidoglycan-degrading property of lysozyme is not necessary for bacterial killing. Confirming the findings of Ibrahim et al., substitution of serine for aspartic acid in the active site of mouse lysozyme M resulted in a complete loss of muramidase activity, and muramidase-deficient recombinant lysozyme readily killed *S. aureus* in vitro^41–43^. It is interesting to note that the absence of muramidase activity had no discernible impact on the capacity of recombinant lysozyme to kill either gram-positive or gram-negative bacteria^18^. It has also been demonstrated that the peptides generated by lysozyme hydrolysis significantly improve its antibacterial effect^44^. Mine^44^ and Pellegrini et al.^45^ found that peptides of amino acid residues (aa) 15–21, 98–108 and 98–112 have an antibacterial effect on gram-negative bacteria such as *Escherichia coli.* With advances in research, antifungal mechanisms of lysozyme have been elucidated^46–48^. In addition to its enzyme activity and cationic nature, studies have shown that the loss of mitochondrial membrane potential, the exposure of phosphatidylserine in the outer leaflets of the cell membrane, chromatin condensation, and DNA damage may all be factors in how lysozyme affects the structure and death of fungi^47,49^. By incubating lysozyme with cell lines, we found that the extracellular fluid after incubation did not exhibit a nucleic acid inhibitory effect, while the intracellular fluid had nucleic acid inhibitory activity. HepG2 is a human-derived hepatic carcinoma cell line with a hypermetabolic enzyme system and powerful secretion function. The pH of in vitro culture liquid is rapidly reduced with cell proliferation (pH < 7)^50^. According to previous study^51–53^, there are two potential ways for lysozyme to pass through the cell membrane in vitro. The first way is receptor-mediated endocytosis by vesicle transportation. The second way is hydrolysis by the extracellular fluid acidic microenvironment. With cell proliferation and metabolite accumulation, the basic amino acid residues of lysozyme are hydrolyzed by the acidic extracellular fluid microenvironment and further degraded into micropeptides or other free active amino acids. These micropeptides or free amino acid uptake is an energy-consuming transport process that is mainly dependent on H^+^ or Ca^2+^ ion concentration conductance. The acidic extracellular fluid microenvironment provides better space for micropeptide absorption. Taken together, we speculate that the second mechanism is the main route for lysozyme absorption under in vitro cell culture conditions, and simultaneously, previous study showed that the acidic environment of artificial gastric juice can hydrolyze lysozyme in vitro^54^. We also noticed that after lysozyme was degraded by gastric acid and bile acid, its ability to inhibit nucleic acid replication was greatly improved compared with that of intact lysozyme. We infer that the degraded lysozyme is released in the form of active micropeptides, which enhance the bacteriostatic and antiviral effects. This is consistent with the findings of Mine et al.^44^.

Since the discovery of the autolysozyme, people have begun to pay attention to the possibility of the antiviral effect of lysozyme^55^. During innate immunity, cationic antimicrobial peptides and proteins can kill microbial and viral pathogens, thereby protecting the host^56^. Lysozyme has cationic activity, and the risk remains low when used in large doses^13^. Our animal experiments also confirmed that when the maximum dose of lysozyme (14 g/kg, equivalent to 500 times the human clinical equivalent dose) was administered to BALB/c mice by oral gavage in a single oral administration, except for the first 3 days after administration, the animals had soft or loose stools, mildly slowed weight gain, and mildly reduced food intake, and no other toxic manifestations were observed. Studies have shown that lysozyme can be used to treat herpes, mumps, chickenpox, hepatitis, influenza, and atypical pneumonia and has been proven to have a potent inhibitory impact on the rabies virus (PRV) and adenovirus^57^. Lysozyme and lactoferrin coadministration has been utilized in other studies to treat bovine viral diarrhea virus, and it was more successful than lysozyme or lactoferrin alone, and the drug efficacy of lysozyme did not weaken over time^12^. The antiviral mechanisms of lysozyme may depend on its cationic protein characteristics^55^. In addition, some studies have found that lysozyme inhibits virus entry by binding with cell receptors or viruses^58^, binding nucleic acids^33^, and inhibiting virus-induced cell fusion. Our research comprehensively observed the effects of lysozyme on different templates and different polymerases. Lysozyme showed a potent and reproducible dose-dependent inhibitory effect on DNA replication, transcription and reverse transcription in vitro, but the difference in inhibitory effect between different templates, such as viruses, prokaryotes, and eukaryotes, was small. However, our in vitro results confirmed a tenfold increase in lysozyme inhibition after the polymerase was replaced in the PCR, so we speculated that lysozyme was more likely to bind to polymerase than to other templates and inhibit nucleic acid replication by inhibiting polymerase activity.

Most amino acids in the body are negatively charged, including some phospholipids on cell membranes. In contrast, there are few positively charged polypeptides in organisms, mainly histones in the nucleus, which generally have specialized physiological functions. Histones have more positive charges and can bind and wrap DNA together to form chromosomes^59^. Early studies have shown that lysozyme can kill HIV, suggesting that lysozyme may interact with DNA and/or RNA. Lysozyme crystal structures were compared to those of histones using computer graphics, which raised the potential that it might have some histone-like characteristics^60^. Lysozyme is also a protein with a relatively high number of positive charges. A short beta-sheet region is surrounded by many helices in the compact tertiary structure of lysozyme, which has a molecular weight of approximately 14,400. The protein is very stable and highly soluble. We analyzed the structure of lysozyme and found that the sequence of lysozyme consists of several relatively concentrated regions, in which a net positive charge is produced by numerous arginine residues^32^. Because of its positive charge, lysozyme can facilitate electrostatic interactions with the viral capsid, negatively charged parts of nucleic acids, and polymerase^15^. Examples include N-acetoxy-2-acetylaminofluorene crosslinking to DNA^61^, sedimentation with DNA^62^, and DNA-membrane interaction^63^. According to the protein domain analysis, we speculate that the catalytic activity center of DNA polymerase I 5'-3' may be the covalent binding site of lysozyme, and DNA polymerase III has the same activity center, where negative charges are concentrated. The positively charged part of lysozyme easily binds with it. Whether there is an antagonistic effect needs further verification. In addition, the α factor of the RNA polymerase I complex is the binding site of DNA, and its active center is aspartic acid. Lysozyme may play its role by affecting the recognition of the α factor and DNA template strand.

In the DNA double-helix structure model proposed by Watson and Crick, the vertical distance between the upper and lower sides of the double-strand after one rotation along the central axis is 3.4 nm, while the vertical distance between the upper and lower adjacent base pairs is 0.34 nm^64,65^. On the one hand, the length of 12–21 amino acids matches an integral multiple of the pitch of the helical structure, so a biologically active polypeptide or local region is generally composed of 7–21 amino acids. On the other hand, from the perspective of cell physiology, some signaling proteins in the organism need guide peptides to connect them to microfilaments or microtubules and then move along the microfilaments or microtubules to specific positions to play a role, so the guide peptides also consist essentially of 7–21 amino acids and are predominantly positively charged. The short-peptide transmembrane hypothesis states that proteins do not easily cross cell membranes and can only enter cells through pinocytosis. However, a short peptide of 7–21 amino acids easily crosses the cell membrane and easily enters the blood through the gastrointestinal tract. Medical short peptides are also 7–21 amino acids; for example, thymosin is often a short peptide of 5–9 amino acids. It would be desirable to administer lysozyme orally to treat systemic diseases; however, the lysozyme in lysosomes is wrapped by membranes, so how to transport this protein across membranes and release it at the desired site is still a difficulty in current research. Although it is unclear how this 14 kDa protein accesses virions or infected cells, lysozyme can reduce the capacity of HIV-1-infected primary T cells and monocytes to shed virus^60^. A synthetic peptide of nine residues extracted from the core region of lysozyme blocks HIV-1 entry at low to moderate nanomolar concentrations. In contrast, the full-length lysozyme was only moderately potent against HIV-1^33^. Although this short peptide has not been found in human tissues or body fluids, proteolysis may occur at the trypsin cleavage site flanking the full-length lysozyme under specific physiological conditions. To verify whether the degraded regions of lysozyme can inhibit nucleic acid replication and screen the active site of lysozyme, HepG2 cell lysate, gastric juice, liver, etc., were used to digest lysozyme. Liver tissue fluid and gastric juice still have the effect of inhibiting nucleic acid replication after lysis; in vivo studies of animal drugs and enteric-coated preparations show that lysozyme still has nucleic acid replication inhibitory activity after being lysed by gastric juice, and it inhibits nucleic acid replication after digestion by jejunal juice. However, the activity was significantly weakened and nucleic acid replication was no longer inhibited after lysis by ileal, colon, rectal, and fecal lavage fluid. The difference may be because pepsin and intestinal trypsin are fixed to the protein cleavage site and retain the active region of lysozyme. However, the cleavage site of the protein by the digestive enzymes secreted in the lower part of the small intestine is not fixed. The digestive enzymes secreted in the lower part of the small intestine are exonucleases, which decompose the protein into amino acids one by one, so the protein is fully inactivated.

According to research, lysozyme not only plays a role in defense mechanisms but also modulates immune responses by promoting and limiting inflammatory responses^22,66^. Most studies on the antitumor effect of lysozyme are based on in vitro studies. Lysozyme releases polynucleotides and induces interferon production^67^. Lysozyme-hydrolyzed peptidoglycan fragments have antitumor activity^68^. Monocytes and macrophages naturally release lysozyme, which may interact with receptor sites on lymphocyte surfaces and help control complicated interactions between monocytes, phagocytes, and lymphocytes as well as lymphocyte activation^69^. The antitumor mechanism of lysozyme can be summarized as direct immune effector activation and indirect host immunity strengthening. The peptides of 8–11 MERS are able to bind to cell-expressed MHC I alleles and then insert into MHC I proteins via MHC I-loading complexes and are expressed on the cell surface^70^. Based on the above, we speculate that the short peptides produced by lysozyme hydrolysis may be active components of antitumor and immune regulation, and the specific mechanism of action remains to be further verified.

## Conclusions

Our study expands the utility of PCR as a screening platform and tool for molecular biology. Lysozyme and its hydrolysate can enter cells and inhibit PCR to varying degrees in vitro. After lysozyme is degraded by gastric acid and bile acid, its ability to inhibit nucleic acid replication and transcription is greatly improved compared with that of complete lysozyme. At the same time, there was no significant difference in the inhibition in the three selected templates, but the inhibition varied widely among different systems of the same template, so we speculated that the inhibition of lysozyme may be related through binding to polymerase.

## Supplementary Information

Supplementary Information 1.

Supplementary Information 2.

## Data Availability

The DNA sequences generated during the current study are available in the NCBI database, NM_01308 (Gene_ID 25617) and NM_005347 (Gene_ID 3309).
