# Supplementary material for: In vitro PCR verification that lysozyme inhibits nucleic acid replication and transcription
Source: Sci Rep. 2023 Apr 19;13:6383. doi: 10.1038/s41598-023-33228-6 (PMC10115842; doi:10.1038/s41598-023-33228-6)
Supplement: Supplementary file 1 — Supplementary Information 1. [file 41598_2023_33228_MOESM1_ESM.pdf]

In vitro PCR verification that lysozyme inhibits nucleic acid replication and transcription

Lu Liu[a,#] Xu Jia[b,#] Xiaoyang Zhao[a,#] Ting Li[a] Ziren Luo[a] Ranxi Deng[a] Bijia Peng[a] Danting Mao[a] Hong Liu[a,*] Qian Zheng[a,*]
[a]Medical Functional Experiment Center, North Sichuan Medical College, Nanchong 637007, People's Republic of China
[b]Department of Pharmacy, Affiliated Hospital of North Sichuan Medical College, Nanchong 637000, People's Republic of China
[#]These authors contributed equally
*Correspondence: zhengqian717693@nsmc.edu.cn ORCID:0000-0002-1754-4851

sFigure 1. Rabbit ileum lavage fluid-treated lysozyme showed no change in EmGfp, Amp and Grp78 DNA replication, transcription or reverse transcription in vitro.

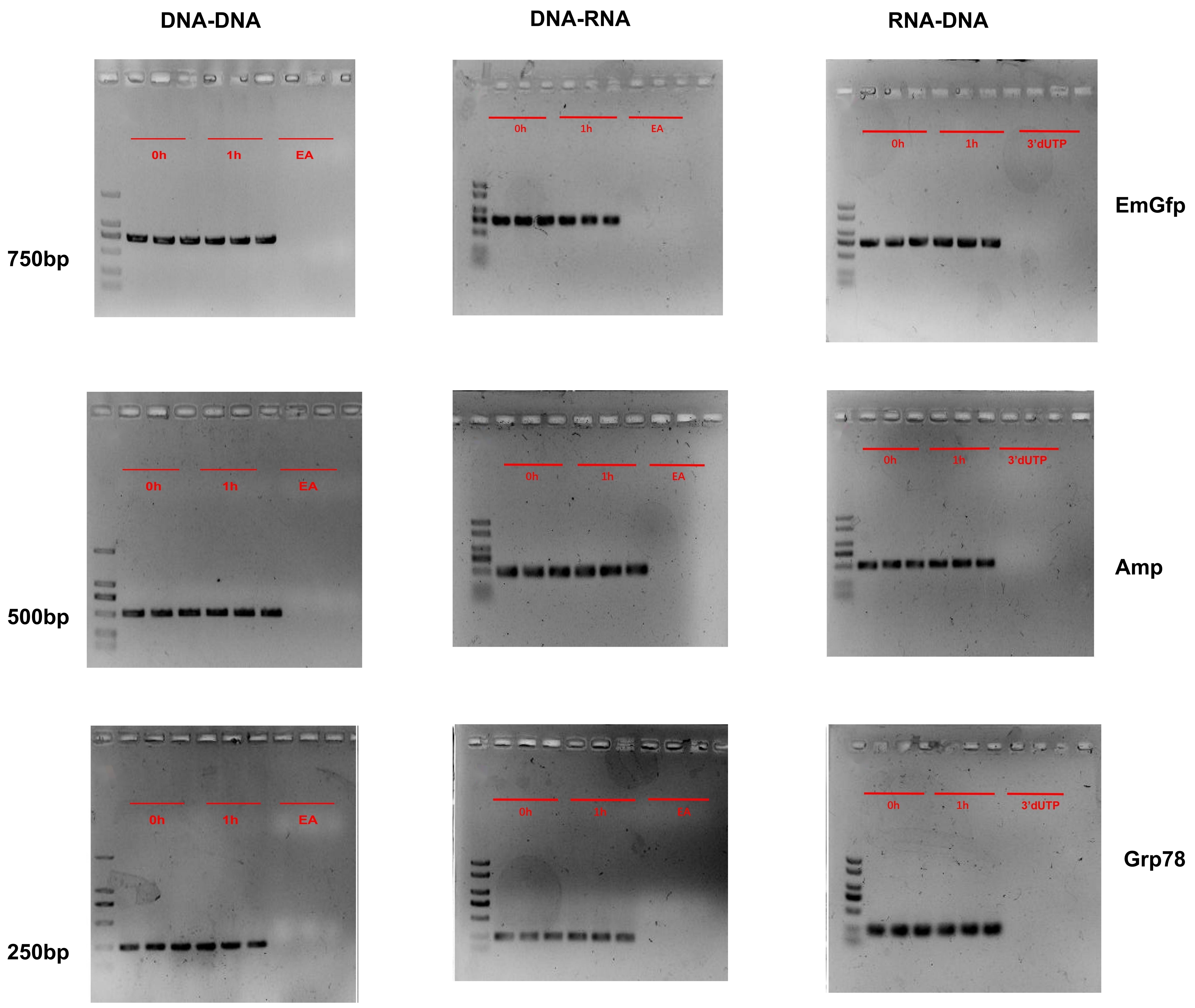

Lysozyme treated with lavage fluid of the upper ileum was added to the reaction system

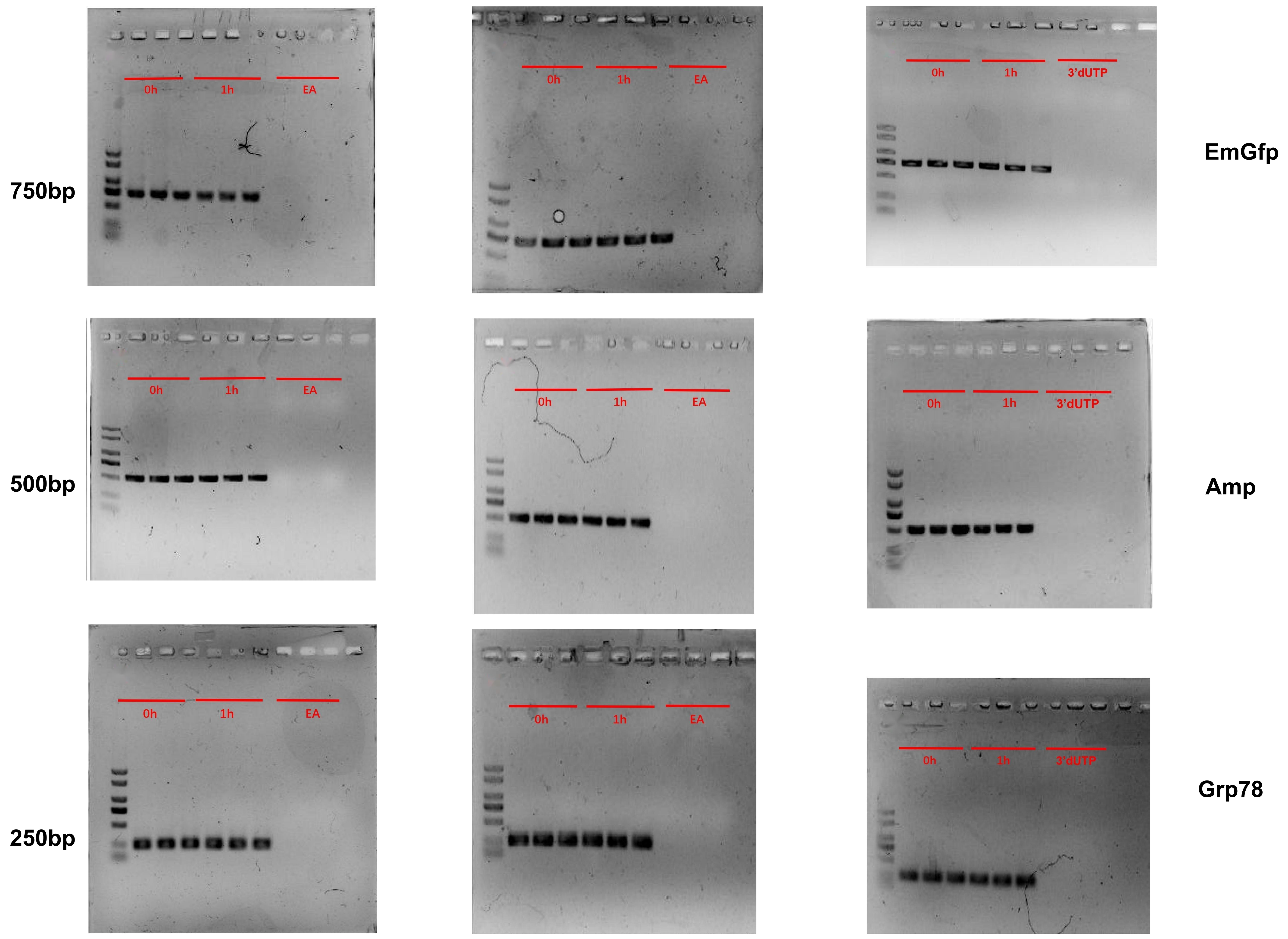

Lysozyme treated with lavage fluid of the lower ileum was added to the reaction system

sFigure 2. Rabbit colon lavage fluid-treated lysozyme did not change EmGfp, Amp and Grp78 DNA replication, transcription or reverse transcription in vitro.

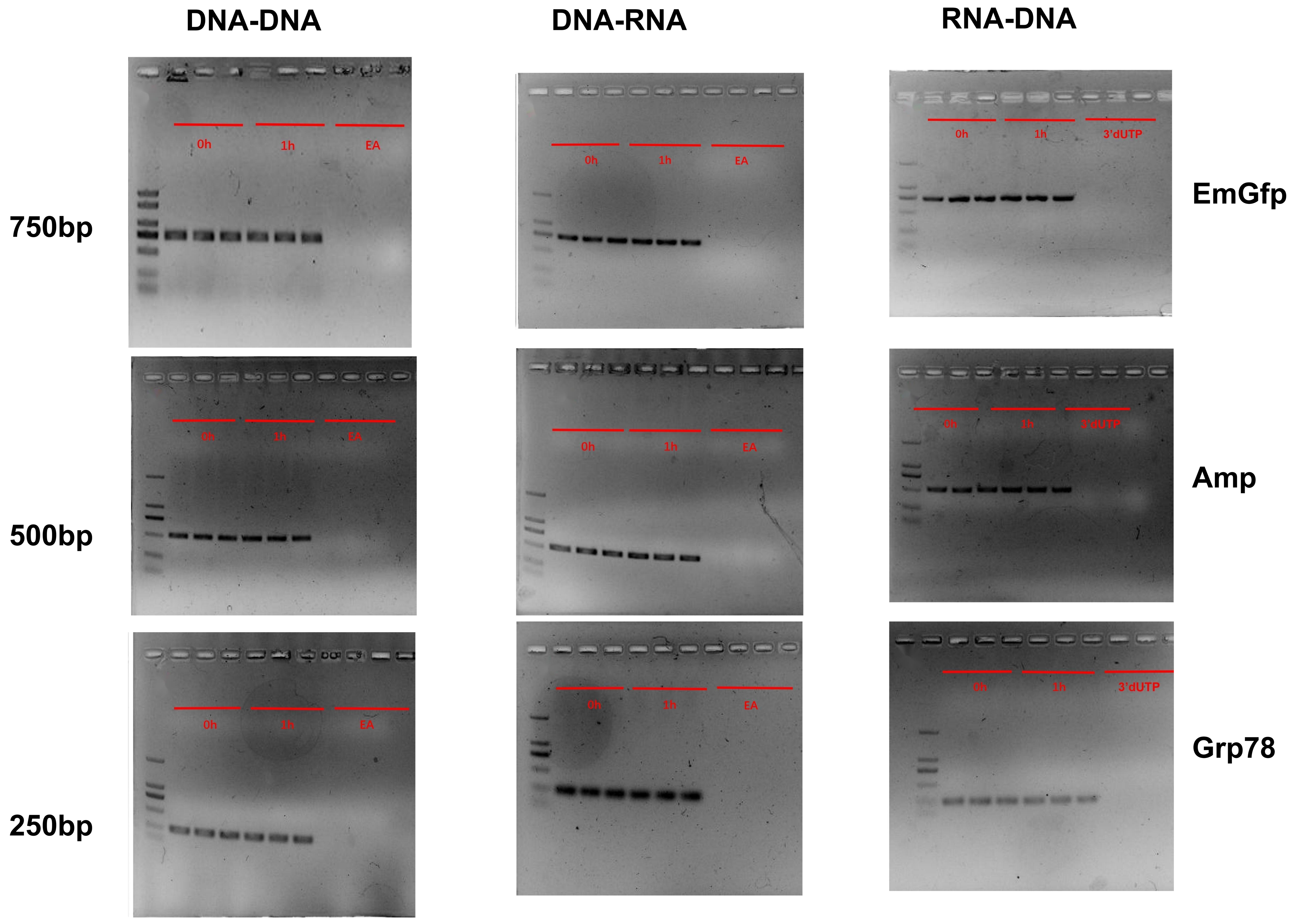

Lysozyme treated with lavage fluid of the upper colon was added to the reaction system

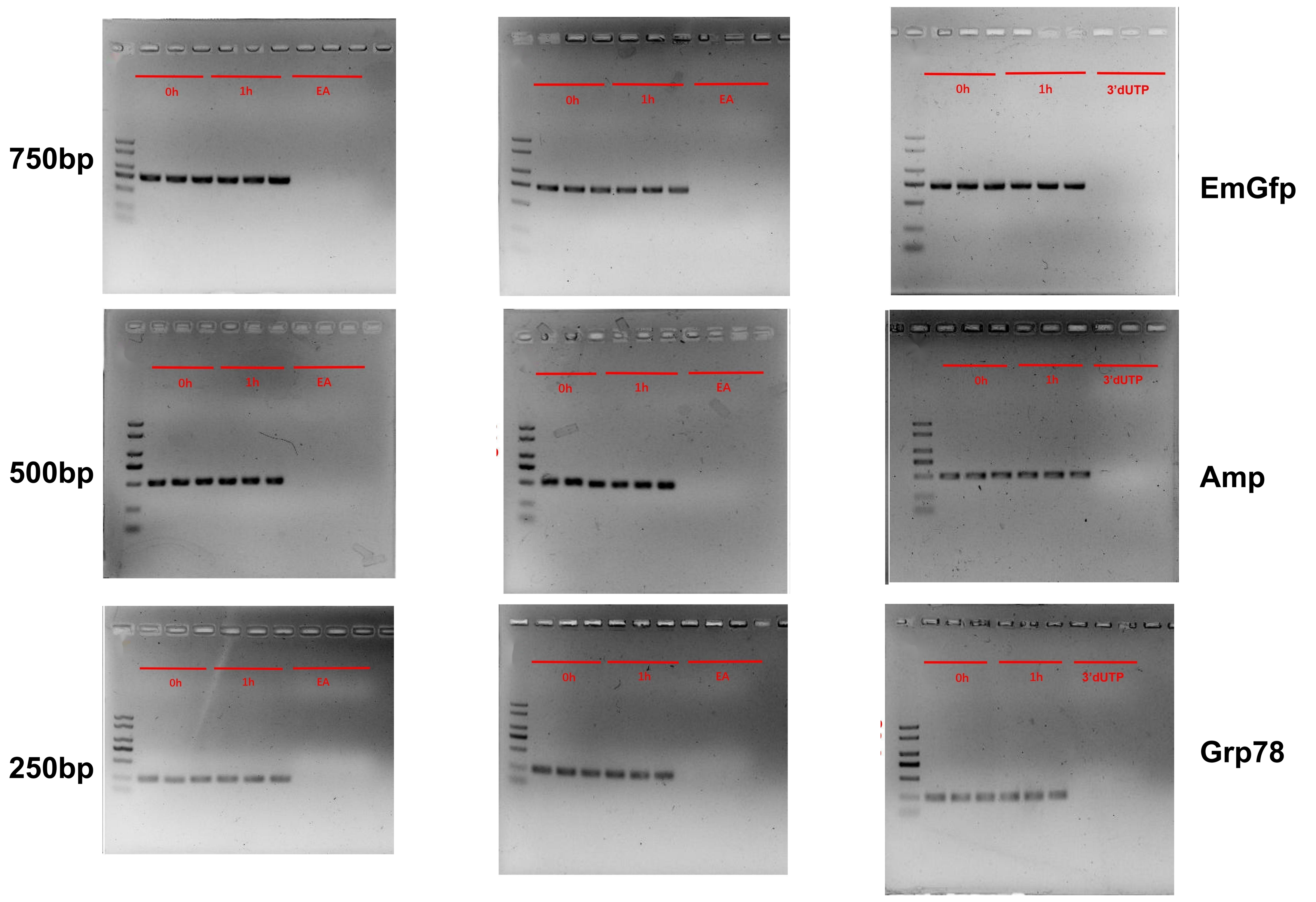

Lysozyme treated with lavage fluid of the middle colon was added to the reaction system

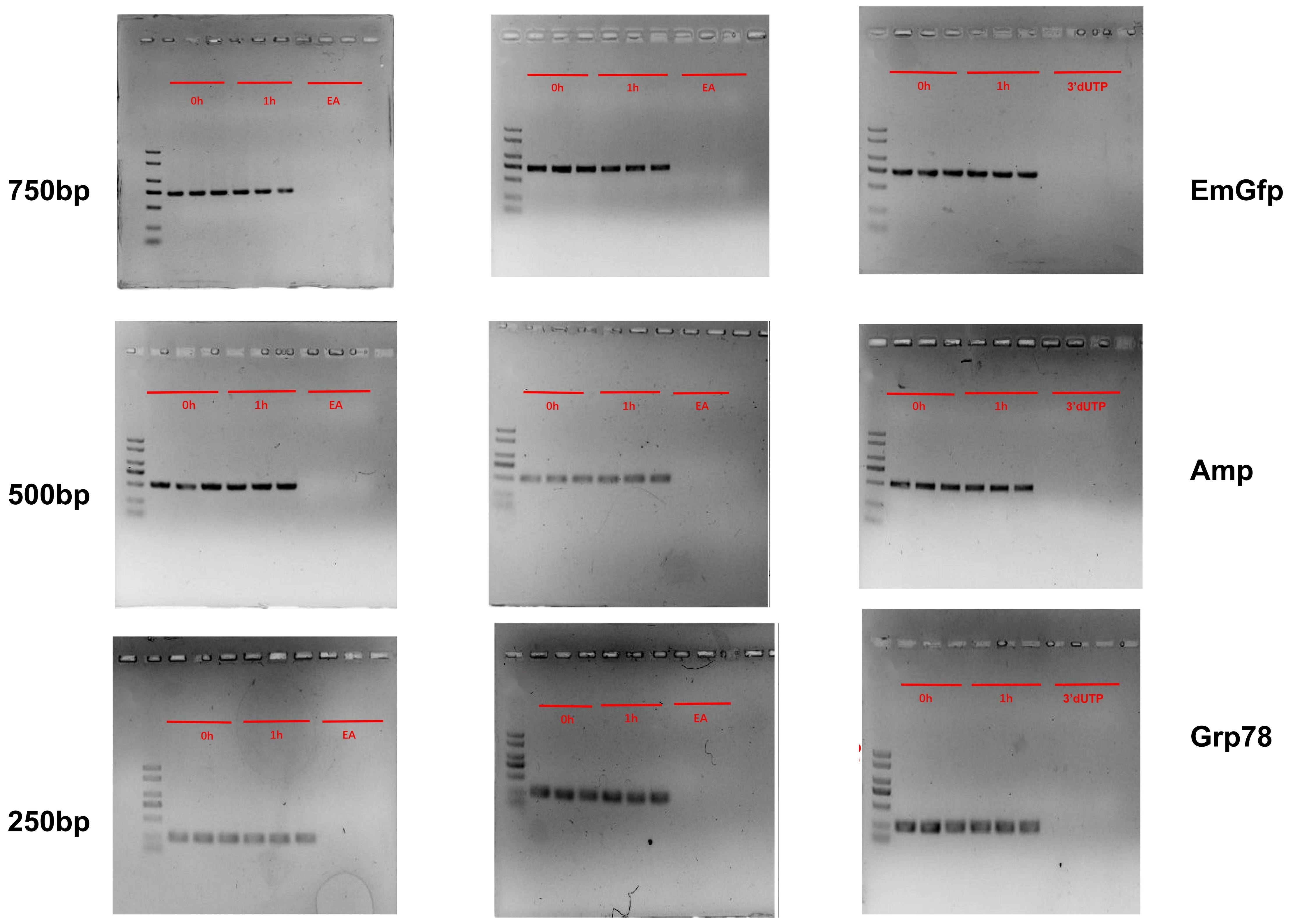

Lysozyme treated with lavage fluid of the lower colon was added to the reaction system

sFigure 3. Rabbit fecal filtrate-treated lysozyme showed no change in EmGfp, Amp and Grp78 DNA replication, transcription or reverse transcription in vitro.

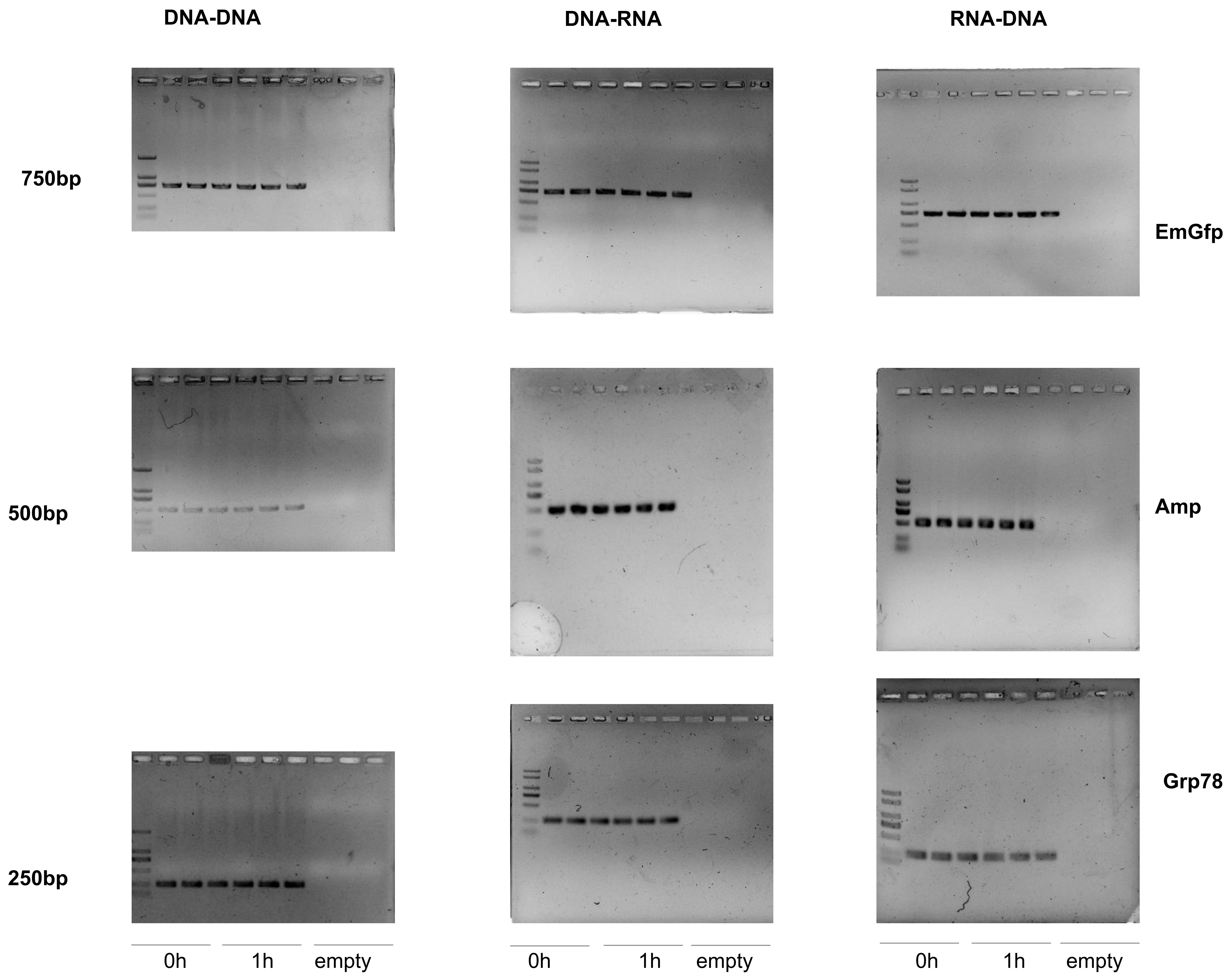

sFigure 4. After lysozyme API gavage, rabbit ileum lavage fluid showed no effect on EmGfp, Amp and Grp78 DNA replication, transcription or reverse transcription in vitro.

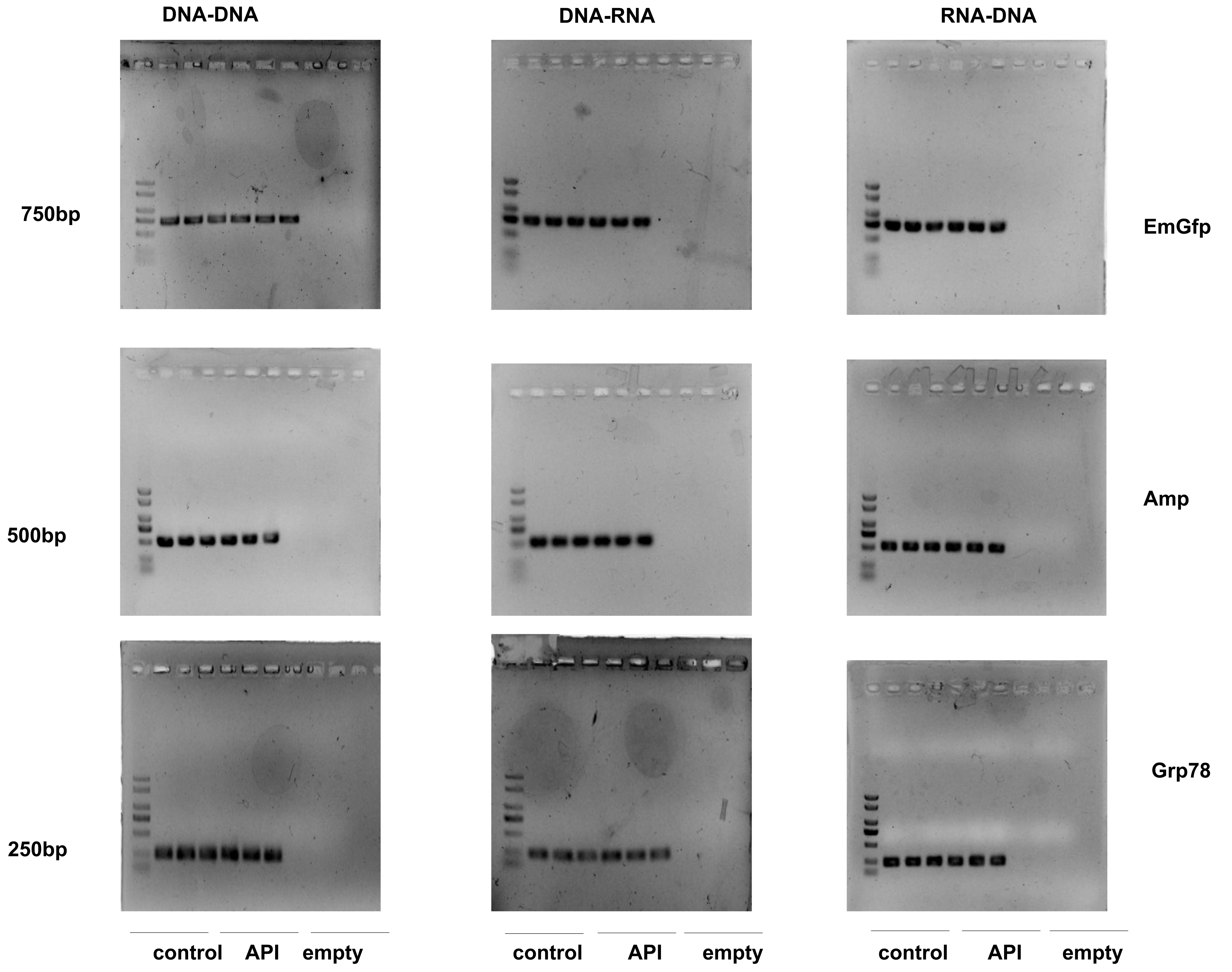

Lavage fluid of the upper ileum was added to the reaction system

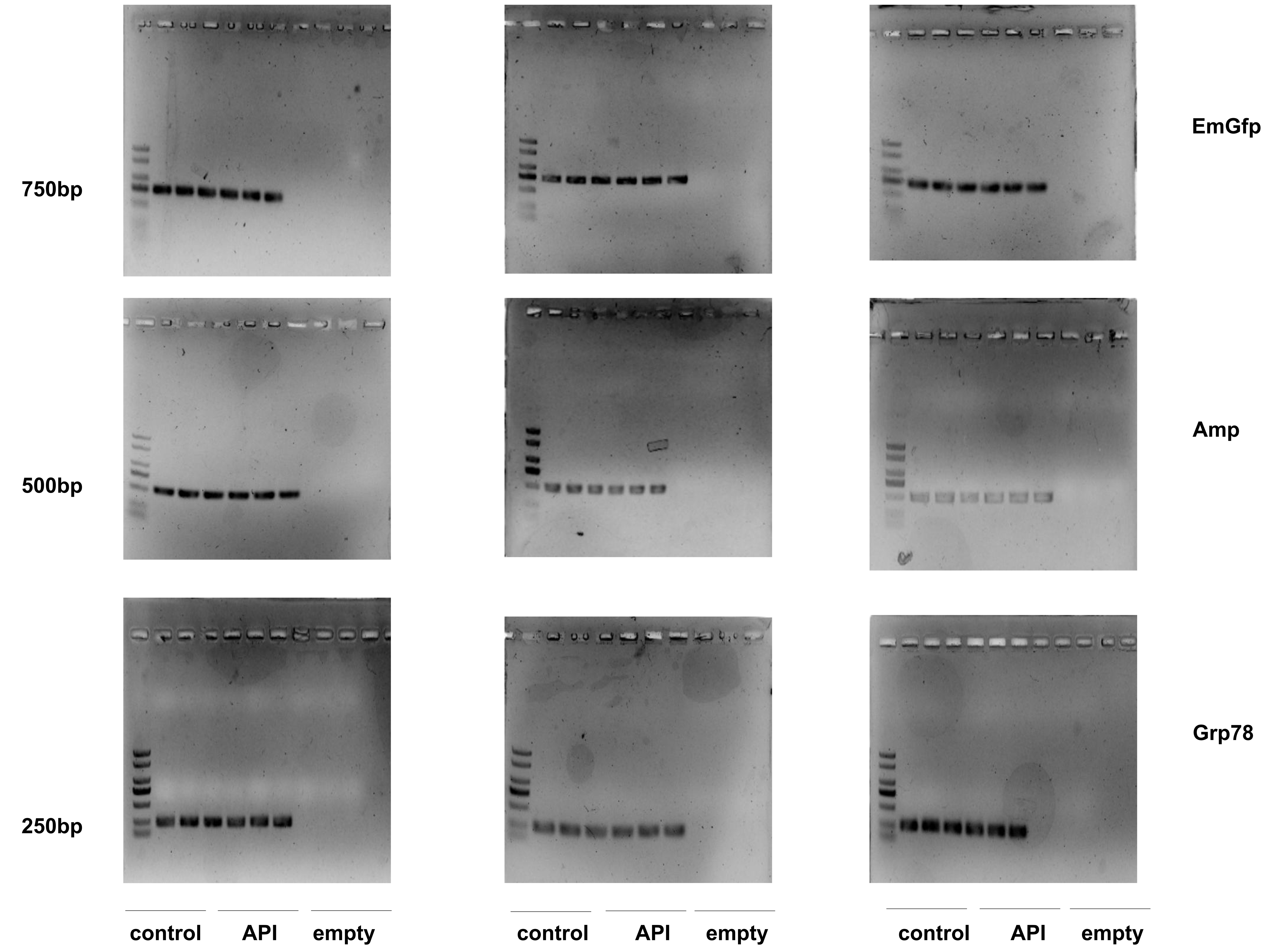

Lavage fluid of the lower ileum was added to the reaction system

sFigure 5. After lysozyme ECT gavage, rabbit ileum lavage fluid showed no effect on EmGfp, Amp and Grp78 DNA replication, transcription or reverse transcription in vitro.

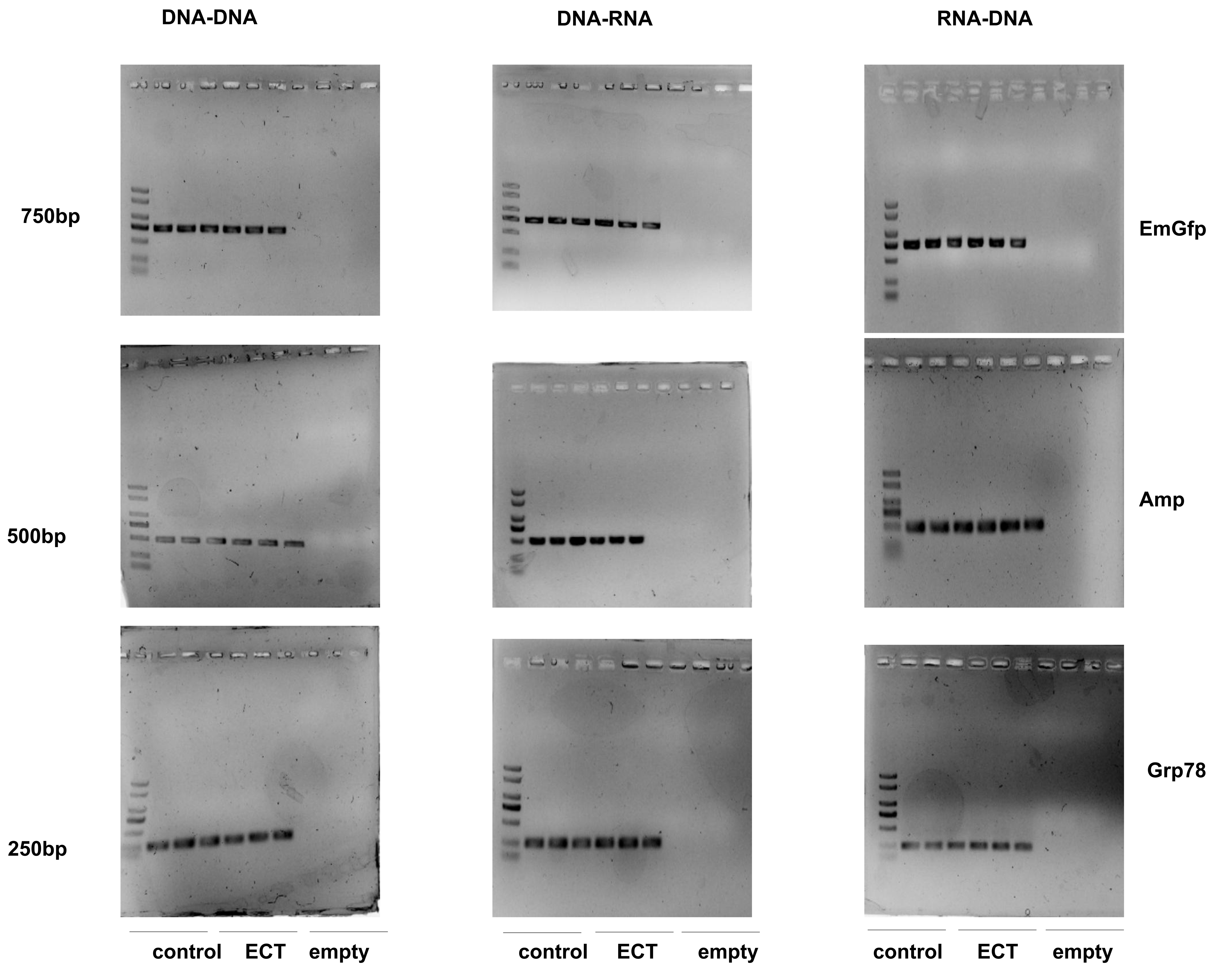

Lavage fluid of the upper ileum was added to the reaction system

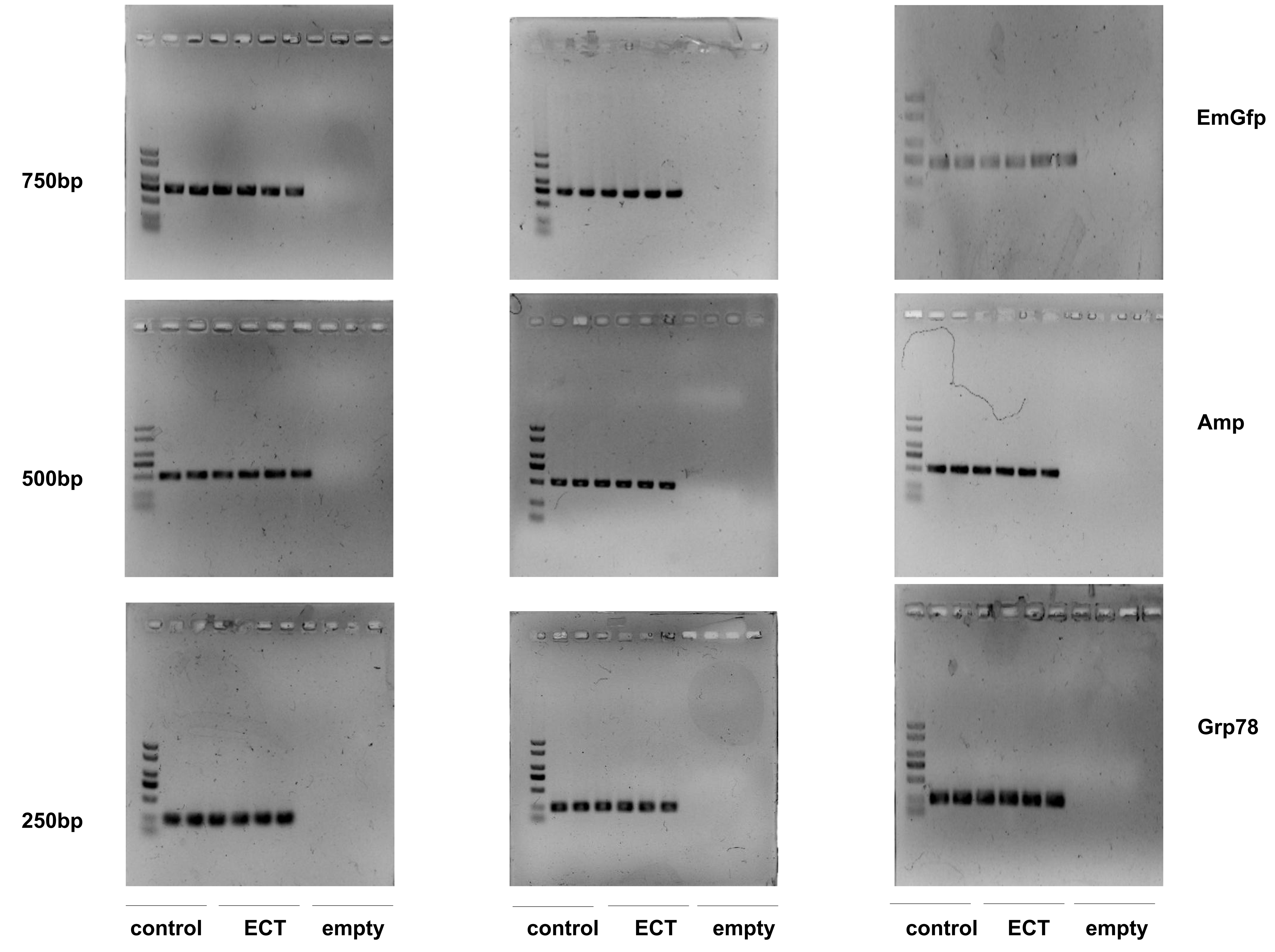

Lavage fluid of the lower ileum was added to the reaction system
