# Supplementary material for: In vitro PCR verification that lysozyme inhibits nucleic acid replication and transcription
Source: Sci Rep. 2023 Apr 19;13:6383. doi: 10.1038/s41598-023-33228-6 (PMC10115842; doi:10.1038/s41598-023-33228-6)
Supplement: Supplementary file 2 — Supplementary Information 2. [file 41598_2023_33228_MOESM2_ESM.doc]

**SUPPLEMENTAL INFORMATION**

**In vitro PCR verification that lysozyme inhibits nucleic acid replication and transcription**

**Lu Liu^a,#^ Xu Jia^b,#^ Xiaoyang Zhao^a,#^ Ting Li^a^ Ziren Luo^a^ Ranxi Deng^a^ Bijia Peng^a^ Danting Mao^a^ Hong Liu^a,*^ Qian Zheng^a^,***

**^a^Medical Functional Experiment Center, North Sichuan Medical College, Nanchong 637007, People’s Republic of China**

**^b^Department of Pharmacy, Affiliated Hospital of North Sichuan Medical**

**College, Nanchong 637000, People’s Republic of China**

**^#^These authors contributed equally**

***Correspondence:** [**zhengqian717693@nsmc.edu.cn**](mailto:zhengqian717693@nsmc.edu.cn) **ORCID:0000-0002-1754-4851**


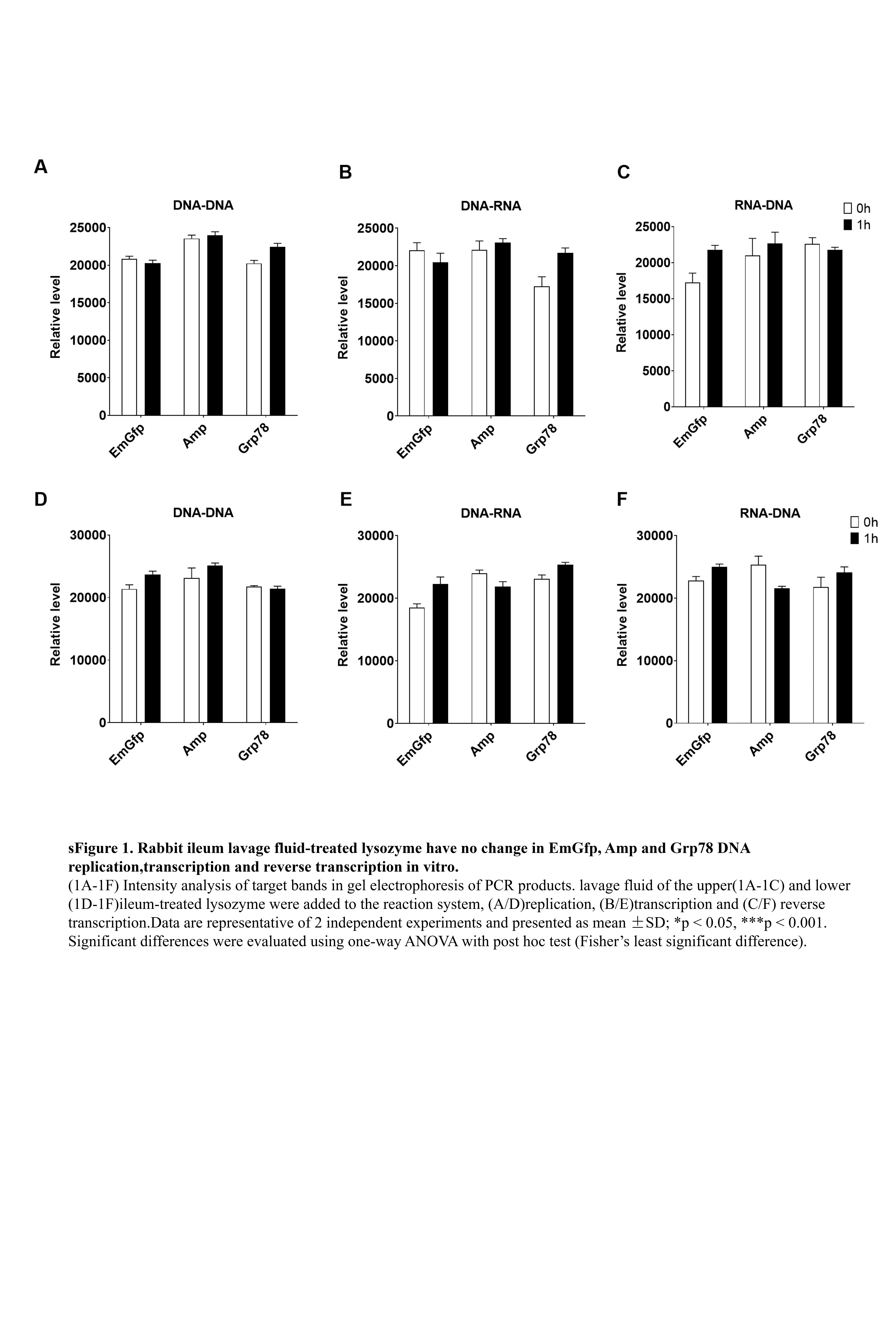


**sFigure 1.** Rabbit ileum lavage fluid-treated lysozyme showed no change in EmGfp, Amp and Grp78 DNA replication, transcription or reverse transcription *in vitro*.

**(1A-1F)** Intensity analysis of target bands in gel electrophoresis of PCR products. Lysozyme treated with lavage fluid of the upper **(1A-1C)** and lower **(1D-1F)** ileum was added to the reaction system: **(A/D)** replication, **(B/E)** transcription and **(C/F)** reverse transcription. Data are representative of 2 independent experiments and presented as the mean ± SD; **p* < 0.05, ****p* < 0.001. Significant differences were evaluated using one-way ANOVA with a post hoc test (Fisher’s least significant difference).


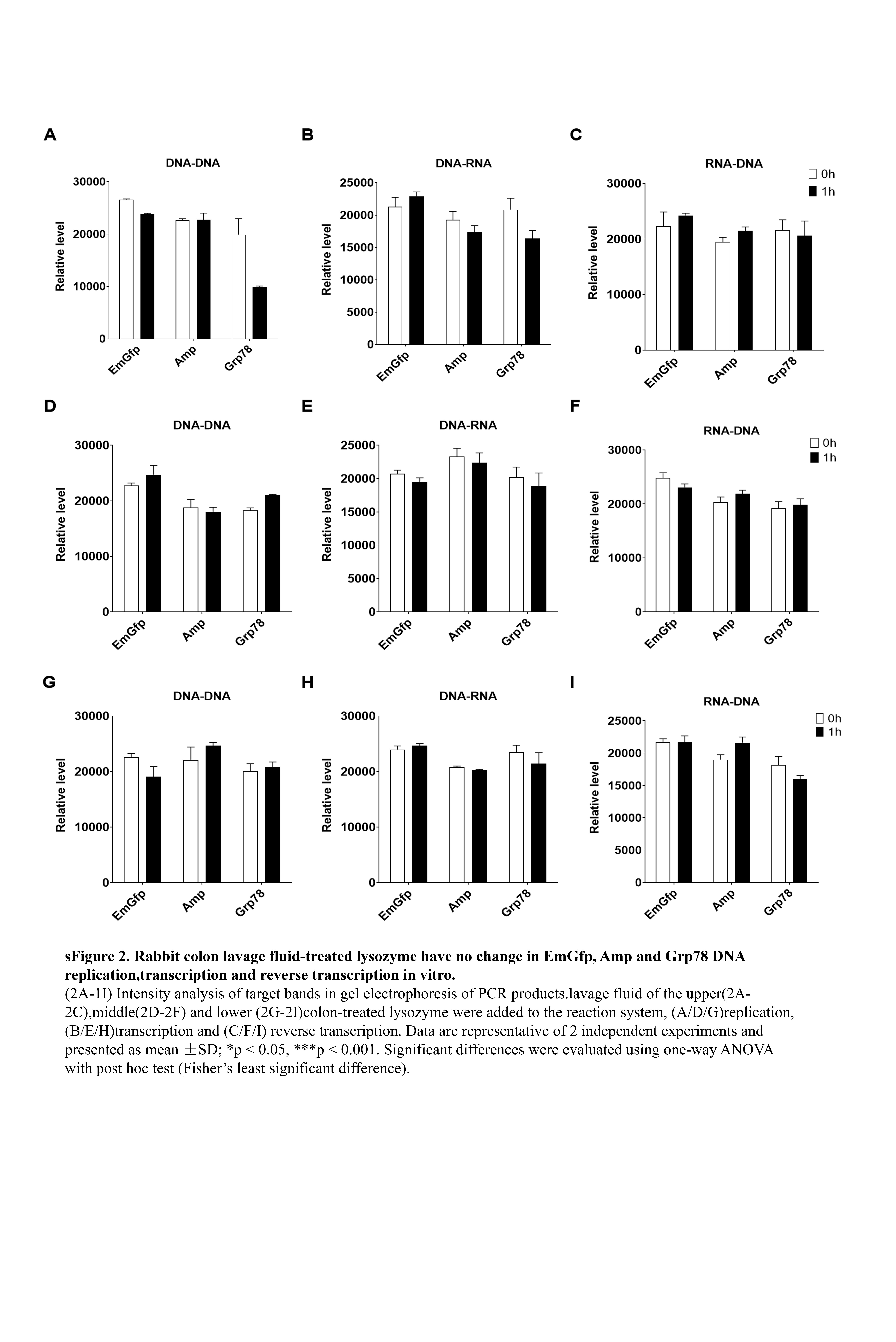


**sFigure 2.** Rabbit colon lavage fluid-treated lysozyme did not change EmGfp, Amp and Grp78 DNA replication, transcription or reverse transcription *in vitro*.

**(2A-2I)** Intensity analysis of target bands in gel electrophoresis of PCR products. Lysozyme treated with lavage fluid of the upper **(2A-2C)**, middle **(2D-2F)** and lower **(2G-2I)** colon was added to the reaction system: **(A/D/G)** replication, **(B/E/H)** transcription and **(C/F/I)** reverse transcription. Data are representative of 2 independent experiments and presented as the mean ± SD; **p* < 0.05, ****p* < 0.001. Significant differences were evaluated using one-way ANOVA with a post hoc test (Fisher’s least significant difference).


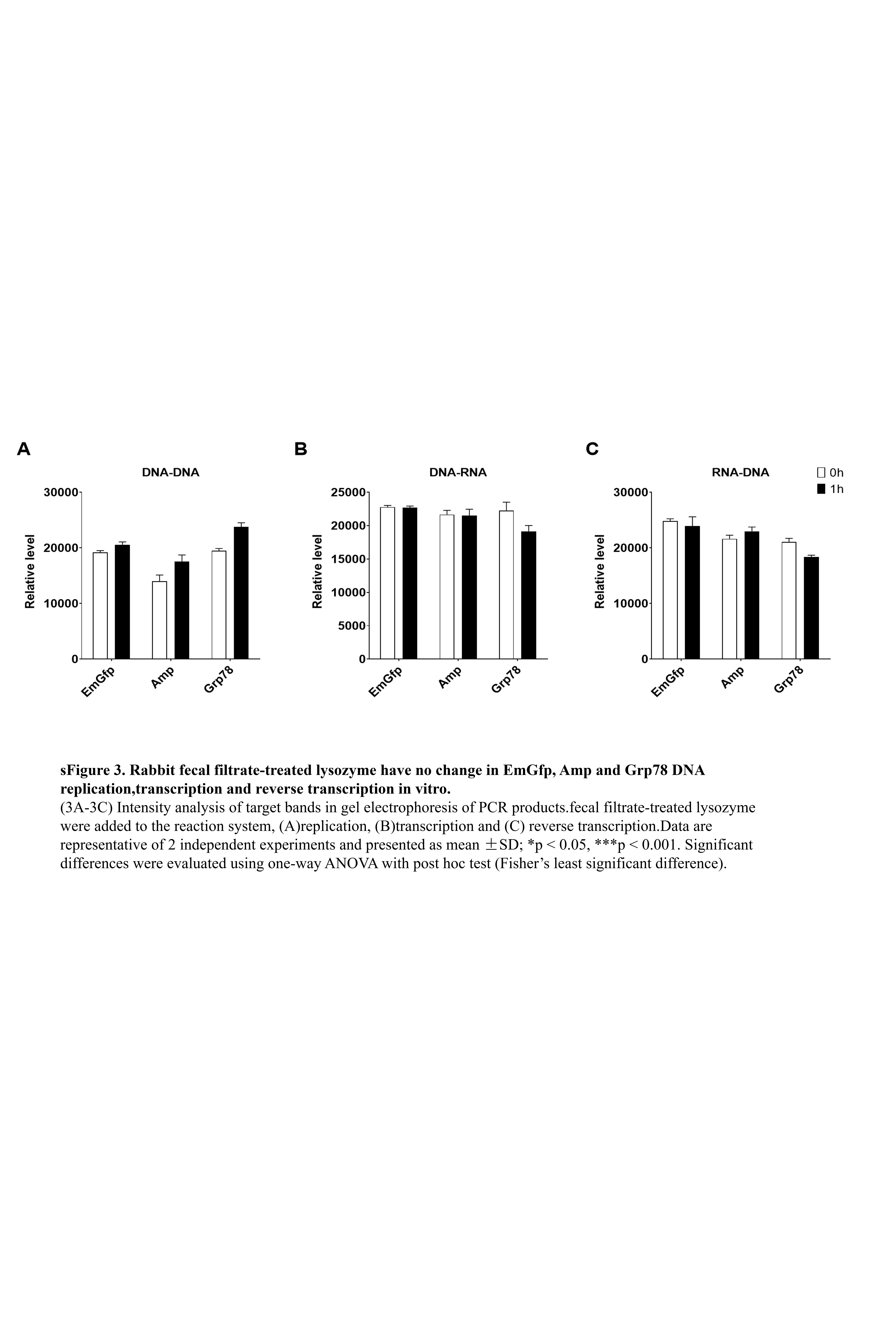


**sFigure 3.** Rabbit fecal filtrate-treated lysozyme showed no change in EmGfp, Amp and Grp78 DNA replication, transcription or reverse transcription *in vitro*.

**(3A-3C)** Intensity analysis of target bands in gel electrophoresis of PCR products. Fecal filtrate-treated lysozyme was added to the reaction system: **(A)** replication, **(B)** transcription and **(C)** reverse transcription. Data are representative of 2 independent experiments and presented as the mean ± SD; **p* < 0.05, ****p* < 0.001. Significant differences were evaluated using one-way ANOVA with a post hoc test (Fisher’s least significant difference).


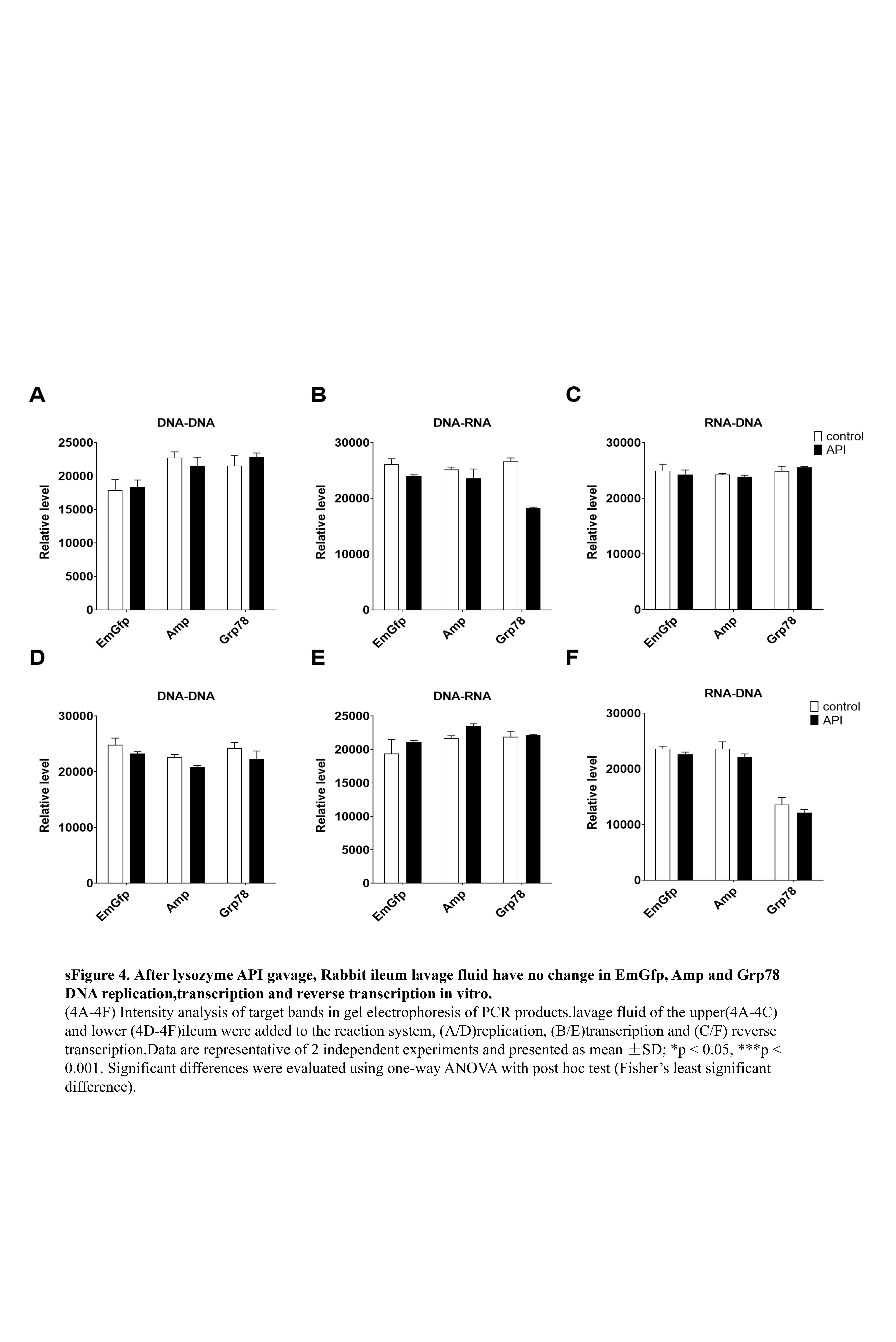


**sFigure 4.** After lysozyme API gavage, rabbit ileum lavage fluid showed no effect on EmGfp, Amp and Grp78 DNA replication, transcription or reverse transcription *in vitro*.

**(4A-4F)** Intensity analysis of target bands in gel electrophoresis of PCR products. Lavage fluid of the upper **(4A-4C)** and lower **(4D-4F)** ileum was added to the reaction system: **(A/D)** replication, **(B/E)** transcription and **(C/F)** reverse transcription. Data are representative of 2 independent experiments and presented as the mean ± SD; **p* < 0.05, ****p* < 0.001. Significant differences were evaluated using one-way ANOVA with a post hoc test (Fisher’s least significant difference).


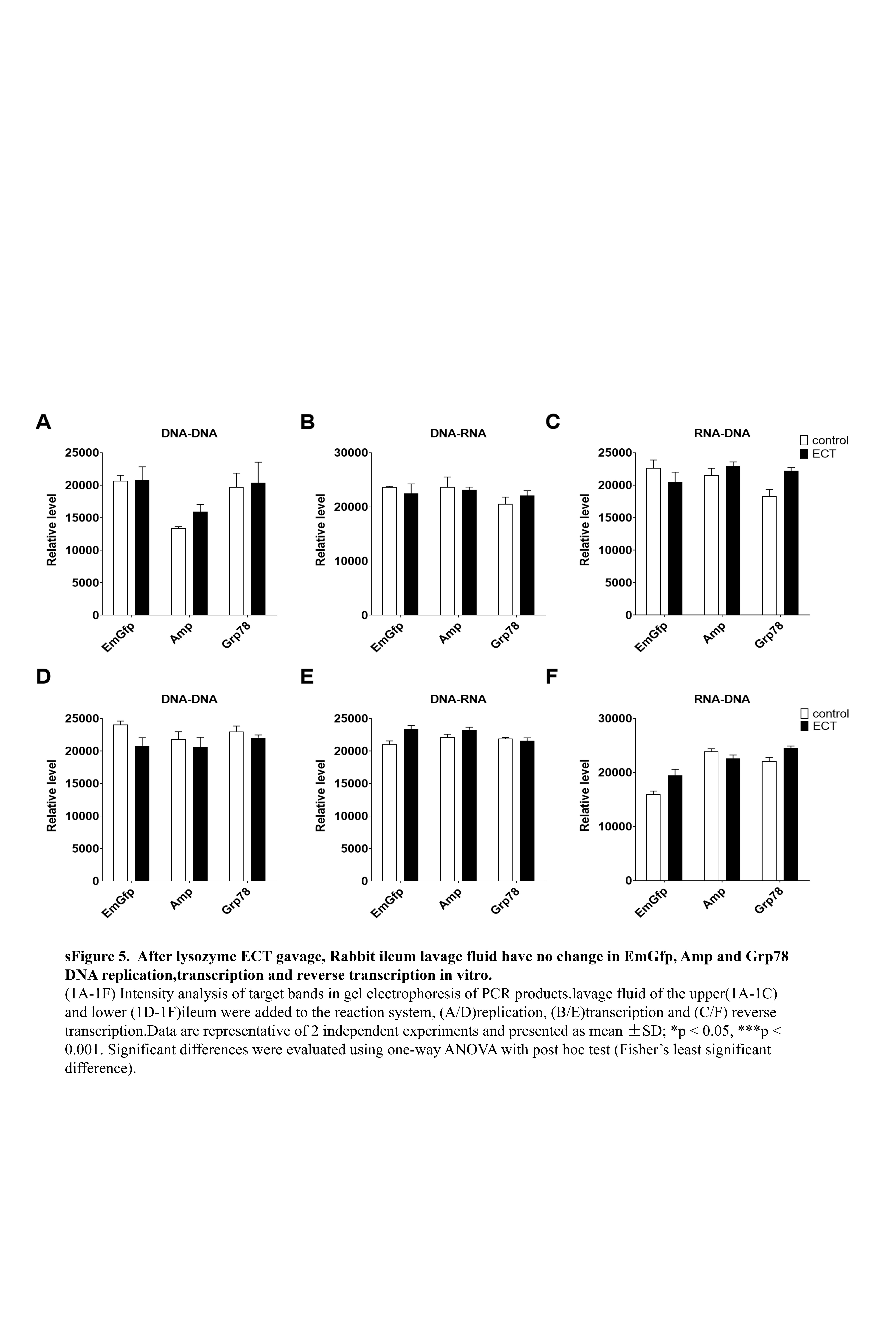


**sFigure 5.** After lysozyme ECT gavage, rabbit ileum lavage fluid showed no effect on EmGfp, Amp and Grp78 DNA replication, transcription or reverse transcription *in vitro*.

**(5A-5F)** Intensity analysis of target bands in gel electrophoresis of PCR products. Lavage fluid of the upper **(5A-5C)** and lower **(5D-5F)** ileum was added to the reaction system: **(A/D)** replication, **(B/E)** transcription and **(C/F)** reverse transcription. Data are representative of 2 independent experiments and presented as the mean ± SD; **p* < 0.05, ****p* < 0.001. Significant differences were evaluated using one-way ANOVA with a post hoc test (Fisher’s least significant difference).
